# Potential distribution of three types of ephemeral plants under climate changes

**DOI:** 10.3389/fpls.2022.1035684

**Published:** 2022-11-23

**Authors:** Zhang Lan, Liu Huiliang, Zhang Hongxiang, Chen Yanfeng, Zhang Lingwei, Kawushaer Kudusi, Dilxadam Taxmamat, Zhang Yuanming

**Affiliations:** ^1^ State Key Laboratory of Desert and Oasis Ecology, Xinjiang Institute of Ecology and Geography, Chinese Academy of Sciences, Urumqi, China; ^2^ University of Chinese Academy of Sciences, Beijing, China; ^3^ Yili Botanical Garden, Xinjiang Institute of Ecology and Geography, Xinyuan, China; ^4^ School of Geography and Tourism, Qufu Normal University, Rizhao, China; ^5^ College of Life Science, Xinjiang Agricultural University, Urumqi, Xinjiang, China

**Keywords:** climate change, ephemeral plant, MAXENT model, potential distribution, species distribution model

## Abstract

**Background:**

Arid and semi-arid regions account for about 40% of the world’s land surface area, and are the most sensitive areas to climate change, leading to a dramatic expansion of arid regions in recent decades. Ephemeral plants are crucial herbs in this area and are very sensitive to climate change, but it is still unclear which factors can determine the distribution of ephemeral plants and how the distribution of ephemeral plants responds to future climate change across the globe.

**Aims:**

Understanding the impact of climate change on ephemeral plant distribution is crucial for sustainable biodiversity conservation.

**Methods:**

This study explored the potential distribution of three types of ephemeral plants in arid and semi-arid regions (cold desert, hot desert, and deciduous forest) on a global scale using the MaxEnt software. We used species global occurrence data and 30 environmental factors in scientific collections.

**Results:**

Our results showed that (1) the average value of the area under the receiver operating curve (AUC) of each species was higher than 0.95, indicating that the MaxEnt model’s simulation accuracy for each species was good; (2) distributions of cold desert and deciduous forest species were mainly determined by soil pH and annual mean temperature; the key factor that determines the distribution of hot desert species was precipitation of the driest month; and (3) the potential distribution of ephemeral plants in the cold desert was increased under one-third of climate scenarios; in the hot desert, the potential suitable distribution for *Anastatica hierochuntica* was decreased in more than half of the climate scenarios, but *Trigonella arabica* was increased in more than half of the climate scenarios. In deciduous forests, the ephemeral plant *Crocus alatavicus* decreased in nearly nine-tenths of climate scenarios, and *Gagea filiformis* was increased in 75% of climate scenarios.

**Conclusions:**

The potential suitable distributions of ephemeral plants in the different ecosystems were closely related to their specific adaptation strategies. These results contribute to a comprehensive understanding of the potential distribution pattern of some ephemeral plants in arid and semi-arid ecosystems.

## Introduction

According to the fifth assessment report of IPCC (Stocker et al., 2013), global warming will continue to intensify in the future. By the end of this century, the global average temperature will increase by 0.3 to 4.8°C, especially in arid and semi-arid regions (Herrera-Pantoja & Hiscock, 2015). Arid and semi-arid areas are where the world’s most irreplaceable biodiversity are located and are an essential component of terrestrial ecosystems, covering approximately 41% of the Earth’s land mass (Chen, 2021). Studies have shown that the arid and semi-arid areas have been expanding over the past 60 years and will continue in the 21st century (Huang et al., 2017), especially in the mid-latitude arid regions, where there are precipitation changes and temperature increases because of climate change, such as reduced precipitation and increased evaporation (Koutroulis, 2019).

Climate change not only significantly affects the land mass of the arid zone but also significantly impacts plant growth and distribution—for example, climate change has effects across multiple plant life stages, seed germination (Mondoni et al., 2012), seedling establishment (Footitt et al., 2018), vegetative growth (Li et al., 2020; Zhao et al., 2021), reproductive development (Hacket-Pain & Bogdziewicz, 2021), and seed dispersal (Hamaoui-Laguel et al., 2015) as well as distribution (Bellard et al., 2012; Hu et al., 2015; Liu et al., 2021). Recently, ecologists recognized species distribution as a common focus of interest because it is the basis for understanding the mechanisms of species formation, evolution, and adaptation. Studies have shown that global climate change leads to plant habitat loss or fragmentation, which significantly threatens the survival of species (Leimu et al., 2010) and leads to the extinction or local extinction of some endangered species (David et al., 2005; Kelly & Goulden, 2008). Detailed reports about the effect of climate change on species are not only essential for understanding species’ origin, distribution, and evolution but also crucial for the conservation of sustainable biodiversity.

Plants have adapted to their environment over long periods and, under specific conditions, have formed a unique group of ephemeral plants. Ephemeral plants are life forms found in dry winter-wet steppes, deserts, and Mediterranean grasslands or areas with a short favorable season (Louise & Bliss, 1982; Liu et al., 2021). Vegetative and reproductive growth quickly takes advantage of optimal conditions, such as temperature, moisture, and sunlight, *etc*. They are gone after a few weeks but leave seeds that will grow into the next generation when conditions become favorable again (Sunmonu & Kudo, 2014). Ephemeral plants are widely distributed in arid and semi-arid ecosystems and are an important component of the nutrient cycle, with an irreplaceable role in stabilizing the sandy surface in deserts—for instance, in the cold deserts, large amounts of snow cover the regions in winter. The snow melts with the warming in early spring, providing a suitable temperature and sufficient moisture for germination and seedling growth of ephemeral plants (Palpurina et al., 2017; Chen et al., 2019). Therefore, temperature and early spring rainfall are essential for ephemeral plant vegetative and reproductive growth. Some ephemeral plants can also survive in hot deserts where there is even more drought and hot than in cold deserts. In the hot desert, extreme diurnal temperature changes occur, with maximum temperatures reaching 40–50°C during summer (Woodell et al., 1986). The total annual precipitation is very low but with large variability in spatial distribution and intensity (Knight & Ackerly, 2003), which provides the living room for some ephemeral plants to survive. However, in deciduous forests, ephemeral plant seeds can germinate, and seedlings grow under a snow cover at lower temperatures (Baskin & Baskin, 1988; Mckenna & Houle, 2000), which enables rapid reproduction before the canopy closes. The ephemeral plant in the deciduous forest usually grows in fertile soil with sufficient soil moisture. Studies also found that the growth of ephemeral plants in deciduous forests is promoted under low temperatures, and increased temperatures are unfavorable for growth (Lapointe, 2001). Thus, with a long period of evolution, ephemeral plants have formed specific adaptive strategies to cope with the survival environment. Meanwhile, precipitation, temperature, and soil are the limiting factors for the growth and distribution of the ephemeral plant, and the suitable habitat will be significantly changed by global climate change. However, which factors determine the distribution of ephemeral plants in a different ecosystem, and how does the distribution of ephemeral plants with special survival strategies respond to future climate change?

Ecological niche modeling has been increasingly used in species distribution, such as invasion species distribution, and species potential distribution under climate change (Phillips et al., 2006; Huang et al., 2018; Ning et al., 2018). This model mainly uses known plant occurrence data and climate variables, according to specific algorithms, constructs a model, and projects the arithmetic consequence to predict the species’ current or potential distribution (Phillips et al., 2006). Since the MaxEnt model has better predictions than the other models under the same conditions, with easy operation, accurate prediction results, and better performance even with incomplete datasets, it has thus been widely used (Negrete et al., 2020; Wang et al., 2020). In addition, MaxEnt models have been used to predict the future potential geographic distribution areas under climate change, such as the distribution of invasion plants—*Prosopis juliflora* and *Ageratina adenophora* (Gu et al., 2021; Amiri et al., 2022), the distribution of some special species—*Impatiens hainanensis* and *Phellodendron amurense* (Huang et al., 2018; Ning et al., 2018), and endangered plant species distribution (Wang et al., 2020; Ye et al., 2021). The MaxEnt model can identify the key factors influencing species distribution and predict distribution changes. Therefore, this approach can be effectively applied to ephemeral plant distribution.

According to the various adaptation strategies of ephemeral plants in cold deserts, hot deserts, and deciduous forests, we hypothesized that (1) the main contributing factors determining the distribution of ephemeral plants may vary in cold desert, hot desert, and deciduous forest ecosystems and (2) under future climate scenarios, the distribution of ephemeral plants in the cold desert, hot desert, and deciduous forest changes significantly. To test this hypothesis, two typical plant species were selected from each ecosystem (cold desert, hot desert, and deciduous forest), and then their distributions were predicted using the MaxEnt model combined with climatic and soil factors under the current and future scenarios.

## Methods

### Environmental variables

A total of 19 bioclimatic and elevation variables were selected, which were downloaded from the WorldClim dataset (http://www.worldclim.org/). Version 2.1 of the WorldClim dataset with a resolution of 30 s was chosen for use in this study, and for the current climate conditions, the average data from 1970 to 2000 were used (Gu et al., 2021). Meanwhile, we selected Model for Interdisciplinary Research on Climate, version 6, to represent future climate scenarios. We chose the data in 2021–2040, 2041–2060, 2061–2080, and 2081–2100 under four shared socioeconomic pathways (ssp): ssp126, ssp245, ssp370, and ssp585 to simulate future climatic scenarios. ssp 126 means lower forcing scenarios (radiative forcing reaches 2.6 W/m^2^ in 2100; the temperature may be lower than 2°C relative to the pre-industrial revolution). ssp245 and ssp370 mean medium forcing scenarios (radiative forcing becomes 4.5 and 7.0 W/m^2^ in 2100). ssp 858 means the highest forcing scenarios (radiative forcing reaches 8.5 W/m^2^ in 2100). According to the model, the temperature may significantly increase at the end of the 21st century, but the precipitation may decrease in arid and semi-arid areas; the trend will become more pronounced as the forcing intensifies. The soil data had a resolution of 30 s and were downloaded from http://soilgrids.org. In addition, we assume that soil conditions do not change in the future climate scenarios and only consider the climatic conditions, as changes in soil properties take place over a long period (Ning et al., 2018) (**Table 1**).

**Table 1:** Total environment variables.

Category		Description	Code
Topography	1	Elevation	
Bioclimate	2	Annual mean temperature (°C)	bio1
	3	Mean diurnal range (°C)	bio2
	4	Isothermality (°C)	bio3
	5	Temperature seasonality (°C)	bio4
	6	Max temperature of warmest month (°C)	bio5
	7	Min temperature of coldest month (°C)	bio6
	8	Temperature annual range (°C)	bio7
	9	Mean temperature of wettest quarter (°C)	bio8
	10	Mean temperature of driest quarter (°C)	bio9
	11	Mean temperature of warmest quarter (°C)	bio10
	12	Mean temperature of coldest quarter (°C)	bio11
	13	Annual precipitation (mm)	bio12
	14	Precipitation of wettest month (mm	bio13
	15	Precipitation of driest month (mm)	bio14
	16	Precipitation seasonality	bio15
	17	Precipitation of wettest quarter (mm)	bio16
	18	Precipitation of driest quarter (mm)	bio17
	19	Precipitation of warmest quarter (mm)	bio18
	20	Precipitation of coldest quarter (mm)	bio19
Soil	21	Bulk density of the fine earth fraction	bdod
	22	Cation exchange capacity of the soil	cec
	23	Volumetric fraction of coarse fragments (>2 mm)	cfvo
	24	Proportion of clay particles (<0.002 mm) in the fine earth fraction	clay
	25	Organic carbon density	ocd
	26	Soil pH	pH
	27	Proportion of sand particles (>0.05 mm) in the fine earth fraction	sand
	28	Soil organic carbon content in the fine earth fraction	soc
	29	Soil classification	
	30	Available water storage capacity	

The strong relation of variables could result in the misinterpretation of the MaxEnt model. Thus, we used ArctoolBox for MaxEnt in Arcgis 10.6 to select variables, and correlation values lower than 0.8 were employed to ensure that the environment is independent (Phillips et al., 2006; Tu et al., 2021). Finally, we obtained 14 environmental variables to build the model for each ephemeral plant (**Table 2**).

**Table 2:** Climatic variables are ultimately used to build the model.

Category	Description		Code
Topography	1	Elevation	
Bioclimate	2	Annual mean temperature (°C)	bio1
	3	Mean diurnal range (°C)	bio2
	4	Isothermality (°C)	bio3
	5	Temperature annual range (°C)	bio7
	6	Annual precipitation (mm)	bio12
	7	Precipitation of driest month (mm)	bio14
	8	Precipitation seasonality	bio15
	9	Precipitation of coldest quarter (mm)	bio19
Soil	10	Proportion of sand particles (>0.05 mm) in the fine earth fraction	sand
	11	Soil pH	pH
	12	Proportion of clay particles (<0.002 mm) in the fine earth fraction	clay
	13	Volumetric fraction of coarse fragments (>2 mm)	cfvo
	14	Bulk density of the fine earth fraction	bdod

### Species occurrence data

**Figure 1:**
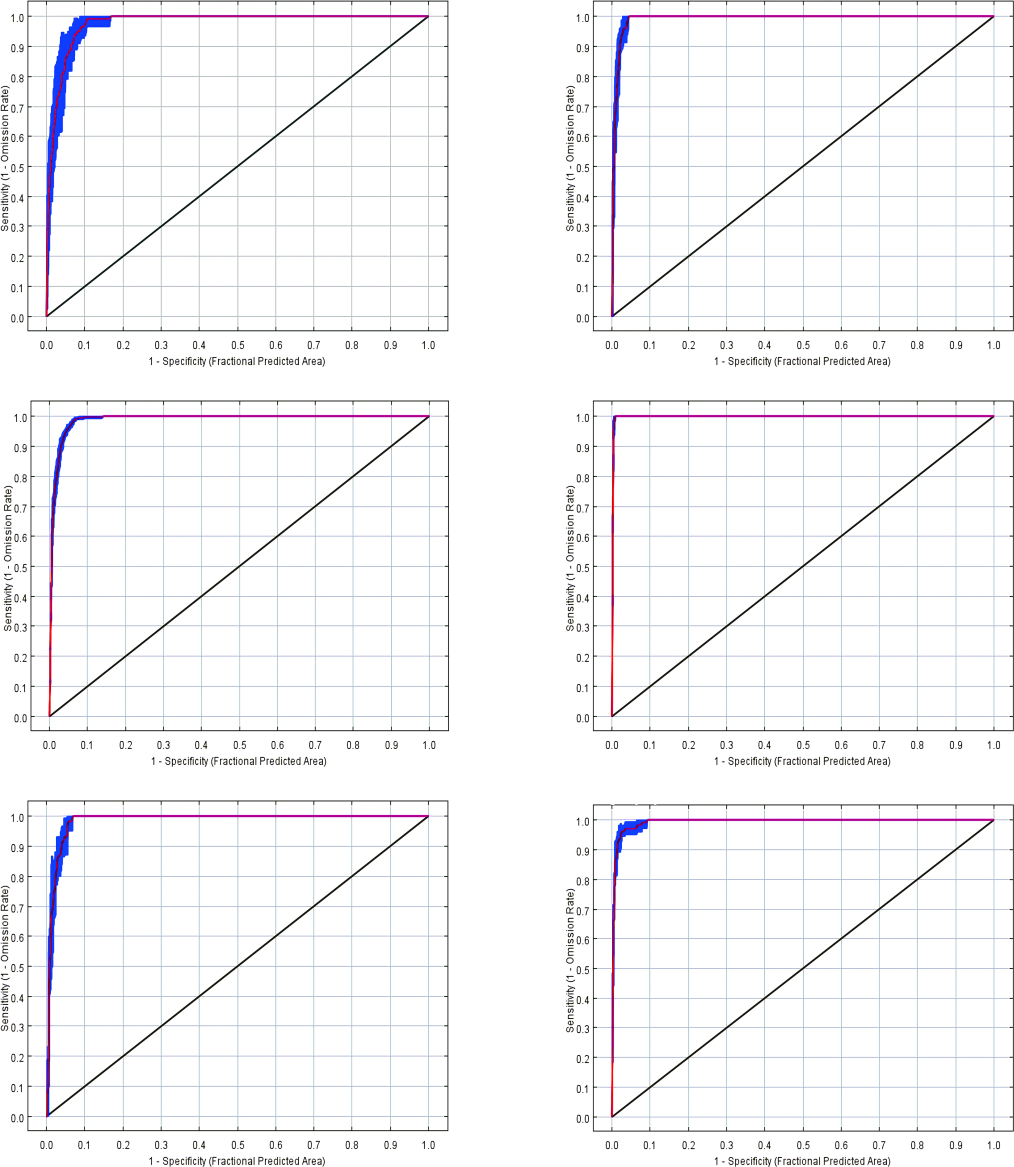
Occurrence records of six ephemeral plants.

In this study, we have selected six ephemeral plants globally, and each species was typical in the ecosystem, with one having a relatively narrow distribution and another one with a relatively wide distribution (**Figure 1**). *Trigonella arcuate* (Fabaceae family) is primarily distributed in Iran, Kazakhstan, Mongolia, China, *etc*. (narrow distribution), and *Tauscheria lasiocarpa* (Brassicaceae) is mainly distributed in Kazakhstan, Pakistan, Iran, Mongolia, Kyrgyzstan, Uzbekistan, Turkmenistan, China, *etc*. (wide distribution). Cold deserts are characterized by cold winters with snowfall, high winter rainfall, and high evaporation over the summer, mainly located in Central Asia. Most plants in cold deserts shed their leaves and have spiny leaves. *Anastatica hierochuntica* (Brassicaceae family) is distributed in Saudi Arabia, Israel, United Arab Emirates, Palestine, Morocco, Algeria, Libya, Jordan, Egypt, Mauritania, and Oman (widely distributed). *Trigonella arabica* (Fabaceae family) is mainly distributed in Israel, Jordan, Palestine, United Arab Emirates (narrow distribution). The two typical plants are distributed in hot desert areas with particular survival strategies. In hot deserts, rainfall is usually deficient and concentrated in short bursts between long rainless periods. Evaporation rates regularly exceed rainfall rates. Sometimes rain starts falling and evaporates before hitting the ground. The hot desert is mainly located in Southern Asia and Africa. Plant leaves in hot deserts with small, thick, and covered in a thick outer layer. *Crocus alatavicus* (Iridaceae) is distributed in Kazakhstan, Uzbekistan, Kyrgyzstan (widely distributed). *Gagea filiformis* distributed in Kazakhstan, Mongolia, Uzbekistan, Kyrgyzstan, and China (narrow distribution). The two typical ephemeral plants were widely distributed in arid and semi-arid deciduous forest areas. Deciduous forest with sufficient rainfall, but the tall trees shed the sunlight for herbaceous plants.

Species occurrence data were downloaded from the Global Biodiversity Information Facility (GBIF) (https://www.gbif.org) and the Chinese Virtual Herbarium (CVH) (http://www.cvh.ac.cn). We used Arcgis 10.6 to test the cross-correlation of 30 variables and only those variables with a correlation coefficient (r^2^) < 0.8 were selected (Negrete et al., 2020; Zhao et al., 2022). Each ephemeral plant’s occurrence records were randomly selected from each cell with dimensions of 20 × 20 km (Boria et al., 2014).

We used the MaxEnt software (3.4.3) (Maxent (amnh.org) to model the six ephemeral plants’ potential distribution (Phillips & Dudík, 2008; West et al., 2016; Abdelaal et al., 2019; Gu et al., 2021; Amiri et al., 2022). We used the receiver operating characteristic to evaluate the MaxEnt model’s performance (West et al., 2016; Abdelaal et al., 2019; Gu et al., 2021). The AUC values range from 0 to 1: AUC >0.9 indicates the highest predictive ability, AUC 0.7–0.9 means good performance, AUC <0.7 indicates that the model is unacceptable (Chen & Chen, 2013; Gu et al., 2021).

This probability was calculated by the occurrence data and randomly selected data with environmental (bioclimatic and soil) variables to generate suitable gradients (Gu et al., 2021; Ye et al., 2021; Amiri et al., 2022). In our study, 75% data were randomly selected to train the model, and 25% data were randomly selected to test each model repeatedly 10 times. A jack-knife test was chosen to evaluate the importance of each variable. In addition, the main environmental variables for each suitability class were extracted from the spatial analysis tool in Arcgis 10.6. (Gu et al., 2021). The probabilities of species distribution were divided into four arbitrary categories with a specific logistic threshold in Arcgis 10.6: unsuitable distribution (0–0.2), lower suitable habitat (0.2–0.4), moderately suitable distribution (0.4–0.6), and highly suitable distribution (0.6–1) (Negrete et al., 2020; Gu et al., 2021; Ye et al., 2021).

To calculate the trends of the potential distribution changes of ephemeral plants in the future, the following equation was used: *Ch*_dis_ *=Fu*_dis_ *- Cu*_dis_.

*Ch*_dis_ means the potential change in distribution between future and current, *Fu*_dis_ means the potential distribution under future climate change scenarios, and *Cu*_dis_ means the potential distribution in current conditions.

## Results

### Model evaluations and the contributions of environmental variables

**Figure 2:**
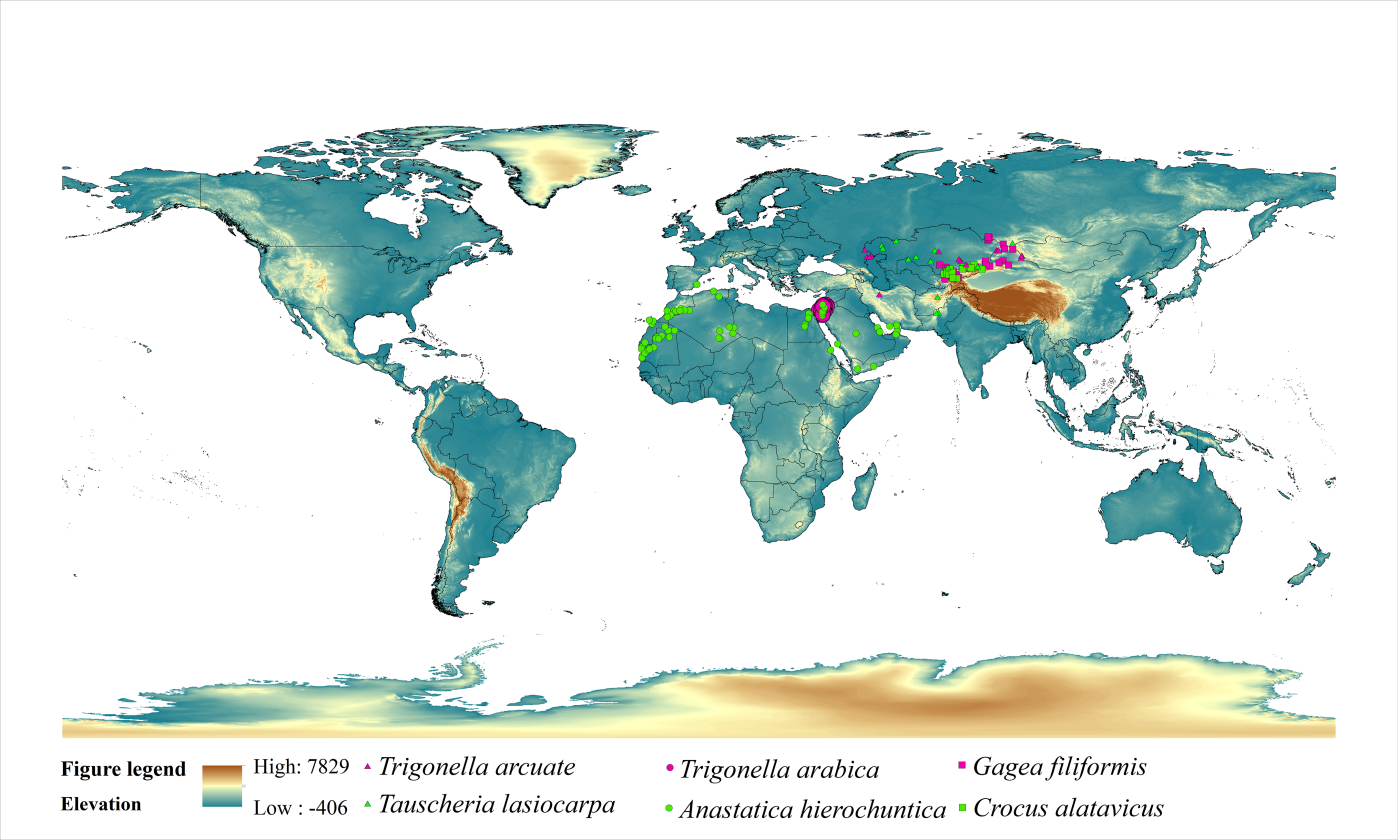
Area under the curve values of six ephemeral plants.

After the model had been built, we obtained the AUC values for *T. arcuata*, *T. lasiocarpa*, *A. hierochuntica*, *T._arabica*, *C. alatavicus*, and *G. filiformis* using the MaxEnt model as 0.977 ± 0.028, 0.985 ± 0.004, 0.984 ± 0.004, 0.995 ± 0.001, 0.977 ± 0.004, and 0.994 ± 0.006, respectively (**Figure 2**), indicating that all models had excellent performance (**Table 3**).

**Table 3:** Species distribution and models’ area under the receiver operating curve (AUC) of six ephemeral plants.

Species	Family	Occurrence records	AUC ± SD	Desert	Countries where mainly distributed
*Trigonella arcuate*	Fabaceae	14	0.977 ± 0.028	Cold desert	Iran, Kazakhstan, Mongolia, Armenia, China
*Tauscheria lasiocarpa*	Brassicaceae	62	0.985 ± 0.004	Cold desert	Kazakhstan, Pakistan, Iran, Mongolia, Kyrgyzstan, Uzbekistan, India, Turkmenistan, China
*Anastatica hierochuntica*	Brassicaceae	254	0.984 ± 0.004	Hot desert	Saudi Arabia, Israel, United Arab Emirates, Palestine, Morocco, Algeria, Libya, Jordan, Egypt, Mauritania, Oman,
*Trigonella arabica*	Fabaceae	477	0.995 ± 0.001	Hot desert	Israel, Jordan, Palestine, United Arab Emirates
*Crocus alatavicus*	Iridaceae	194	0.977 ± 0.004	Deciduous forest	Kazakhstan, Uzbekistan, Kyrgyzstan
*Gagea filiformis*	Liliaceae	58	0.994 ± 0.006	Deciduous forest	Kazakhstan, Mongolia, Uzbekistan, Kyrgyzstan, China

#### Cold desert

From the MaxEnt model, the major contributing variables to *T. arcuate* (**Table 4**) were as follows: pH (35.7%), annual mean temperature (19.8%), precipitation seasonality (coefficient of variation) (10.2%), isothermality (Bio 2/Bio 7) (10.2%), and bulk density of the fine earth fraction (8.7%). The potential suitability of *T. lasiocarpa* (**Table 4**) was influenced by the following: pH (37.2%), annual mean temperature (28.3%), annual precipitation (7.6%), precipitation seasonality (coefficient of variation) (7.3%), and precipitation of the coldest quarter (4.7%). Among the variables, soil pH made the largest contribution to the potential suitability of *T. arcuata* and *T. lasiocarpa* in our model (35.7% and 37.2%); the annual mean temperature was the second most influential factor (19.8% and 28.3%).

**Table 4:** Contribution of environmental factors to the distribution of ephemeral plants in cold deserts.

Species	Environment factors	Range	Contribution rate	Importance value
*Trigonella arcuate*	pH	7.66–9.63	35.7%	0.1%
	Bio1	-0.56–12.06	19.8%	5.5%
	Bio15	0–43.37	10.2%	5.6%
	Bio3	13.50–28.26	10.2%	40.7%
	Bdod	132.46–180.6	8.7%	36.9%
	Sand	-74.90–328.72	8.5%	0.5%
	Bio2	7.19–11.58	2.9%	0.2%
*Tauscheria lasiocarpa*	pH	7.51–9.52	37.2%	3.5%
	Bio1	1.13–12.85	28.3%	14.3%
	Bio12	-573.50–300.51	7.6%	52.6%
	Bio15	0–48.35	7.3%	3.7%
	Bio19	15.2 –147.44	4.7%	10%
	Bdod	128.02–181.9	4.5%	7.3%
	Sand	-85.30–359.42	4.3%	0.3%

The suitable variable ranges of *T. arcuate* were observed (**Table 4**) as follows: pH (7.66–9.63), annual mean temperature (-0.56–12°C), and precipitation seasonality (-18.47–43.37). The suitable variable ranges for *T. lasiocarpa* potential were also observed as follows: pH (7.51–9.52), annual mean temperature (1.13–12.85°C), and annual precipitation (ranges from 0 - 229.74 mm). According to the ManEnt model, pH and annual mean temperature had a strong effect on the potential suitability of *T. arcuate* and *T. lasiocarpa*, and the two species have preferred distribution in the annual mean temperature ranges lower than 12°C and in alkaline soil (**Table 4**).

#### Hot desert

The model results indicated that the major variables of *A. hierochuntica* were as follows (**Table 5**): precipitation of driest month (35.3%), annual precipitation (18%), precipitation of coldest quarter (11%), annual mean temperature (9.7%), and coarse fragment volumetric total (5.2%). The suitable variable ranges for *T. arabica* potential were observed: precipitation of driest month (47.5%), precipitation of coldest quarter (21.2%), mean diurnal range (10.9%), sand (6.6%), and annual mean temperature (5.9%). Among the variable types, precipitation of the driest month made the largest contribution of *A. hierochuntica* and *T. arabica* in our model (35.3% and 47.5%, respectively).

**Table 5:** Contribution of environmental factors to the distribution of ephemeral plants in hot deserts.

Species	Environment factors	Range	Contribution rate	Importance value
*Anastatica hierochuntica*	Bio14	0–0.28	35.3%	0.9%
	Bio12	15.31–112.93	18%	63.5%
	Bio19	2.56–21.76	11%	7.6%
	Bio1	18.14–24.02	9.7%	4.7%
	Cfvo	230.71–608.3	5.2%	2%
	Bio2	11.69–14.48	4.2%	1.5%
	Bio7	25.24–32.78	4.1%	4.6%
*Trigonella arabica*	Bio14	<0	47.5%	5.5%
	Bio19	111.43–442.92	21.2%	1.1%
	Bio2	10.54–12.81	10.9%	0.4%
	Sand	-62.20–285.21	6.6%	10.1%
	Bio1	17.37–21.23	5.9%	0.7%
	Bio7	23.55–29.07	2.5%	51.9%
	Elev	0–1325.04	1.8%	0.5%

From the results of *A. hierochuntica*, the suitable precipitation of the driest month (0–0.28 mm), annual precipitation (15.31–112.93 mm), and precipitation of the coldest quarter (2.56–21.76 mm) were observed. The suitable variable ranges for *T. arabica* potential were observed as follows: the suitable precipitation of the driest month was lower than 0 mm, the precipitation of the coldest quarter was 111.43–442.92 mm, and the mean diurnal range was 10.54–12.81 mm. The model’s results show that the precipitation of the driest month was the main contributing factor to the distribution of *A. hierochuntica* and *T. arabica*, respectively, and the two species’ potential distribution was mainly limited by precipitation (**Table 5**).

#### Deciduous forest

The model showed that the major variables of *C. alatavicus* were as follows: annual mean temperature which ranges from -1.82 to 10.45°C (contributes 27.2%), elevation which ranges from 1,246.18 to 5,457.36 m (contributes 19.6%), sand which ranges from -80 to 343.94 (contributes 16.3%), precipitation seasonality which ranges from 24.99 to 59.94 (contributes 15.6%), and Bdod which ranges from 119.91 to 183.7 (contributes 9.4%) (**Table 6**).

**Table 6:** Contribution of environmental factors to the distribution of ephemeral plants in deciduous forests.

Species	Environment factors	Range	Contribution rate	Importance value
*Crocus alatavicus*	Bio1	-1.82–10.45	27.2%	4.7%
	Elev	1,246.18–5,457.36	17.1%	5%
	Sand	-80–343.94	16.3%	2.4%
	Bio15	24.99–59.94	15.6%	27.7%
	Bdod	119.91–183.7	9.4%	1.3%
	Bio14	7.7–31.81	5.6%	13.1%
	Bio3	25.59–39.98	2.8%	26.6%
*Gagea filiformis*	pH	6.45–7.32	23.3%	20.3%
	Bio1	-1.35–9.3	18.6%	4.5%
	Elev	1,160.16–3,973.81	17.8%	5.1%
	Bio19	75.69–195.59	15.7%	1.4%
	Bio7	35.87–46.66	7.6%	0.1%
	Bio2	11.14–13.27	5.2%	0.8%
	Bio14	9.68–30.34	3.1%	2.2%

The potential suitability of *G. filiformis* were as follows: pH which ranges from 6.45 to 7.32 (contributes 23.3%), annual mean temperature which ranges from -1.35 to 9.3°C (contributes 18.6%), elevation which ranges from 1,160.16 to 3,973.81 m (contributes 17.8%), precipitation of coldest quarter which ranges from 75.69 to 195.59 (contributes 15.7%), and temperature annual range from 35.87 to 46.66 (contributes 7.6%) (**Table 6**). The results show that the soil properties and annual mean temperature mainly contributed to the distribution of *C. alatavicus and G. filiformis*. The temperature and soil pH limited the two species’ distribution in deciduous forests (**Table 6**).

### Current distribution of ephemeral plants

**Figure 3:**
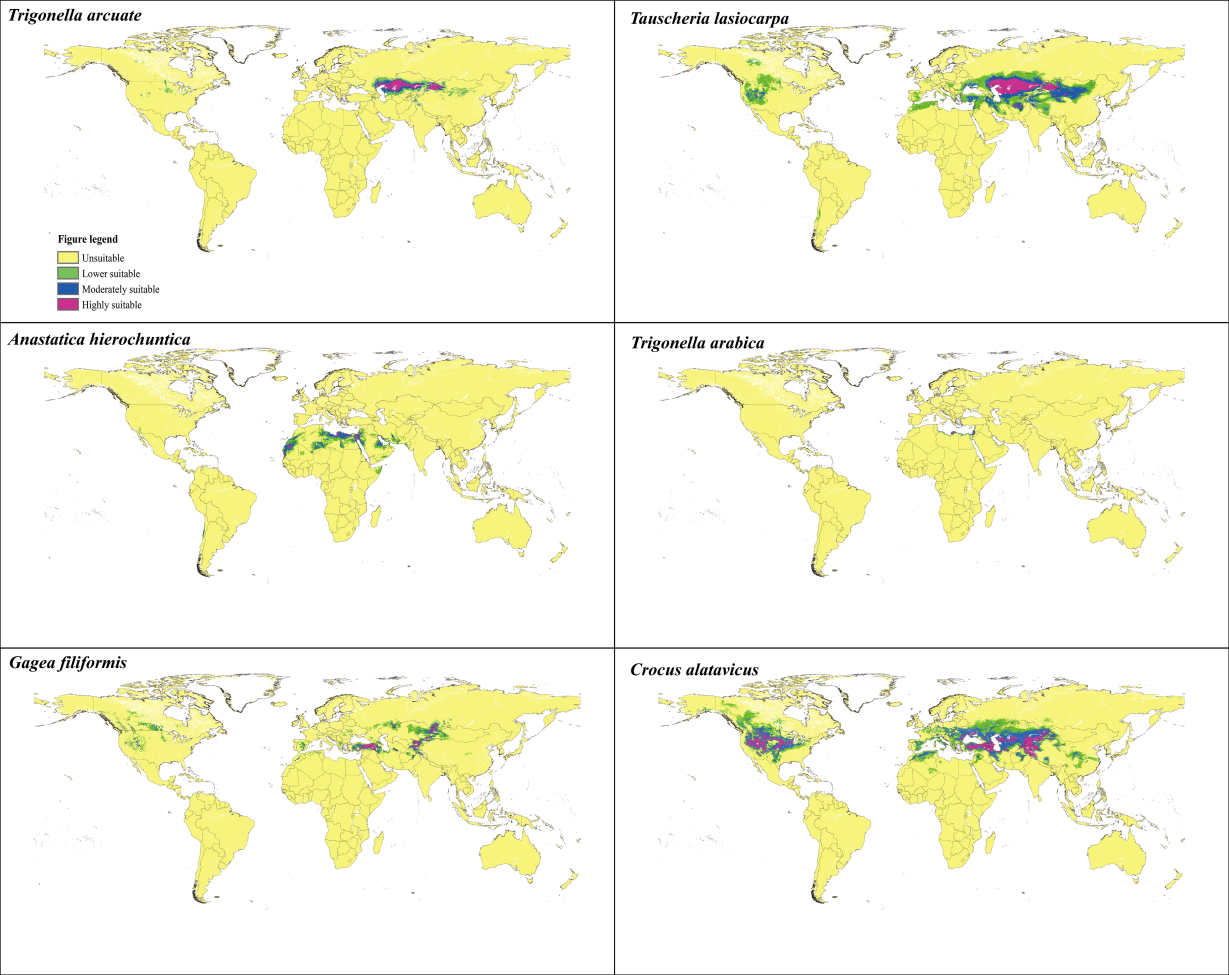
Potential suitable distribution habitat of six ephemeral plants under the current scenarios.

**Table 7** shows the percentages of different suitable living areas under the current condition. According to the potential suitable areas of *T. arcuata*, *T. lasiocarpa*, *A. hierochuntica*, *T._arabica*, *C. alatavicus*, and *G. filiformis* (**Figure 3**), the current total potential suitable distribution areas were 448.78 × 10^4^, 1,802.66 × 10^4^, 448.45 × 10^4^, 31.84 × 10^4^, 2,031.45 × 10^4^, and 557.14 × 10^4^ km^2^, respectively. The highest suitable habitat occupied 29.09, 155.32, 46.51, 3.56, 41.50 × 10^4^, and 60.49 × 10^4^ km^2^, while for the moderately suitable habitat, these values were 65.49 × 10^4^, 255.46 × 10^4^, 155.32 × 10^4^, 9.61 × 10^4^, 65.70 × 10^4^, and 112.52 × 10^4^ km^2^ (**Table 7**).

**Table 7:** Current potential suitable distribution area of six ephemeral plants (×10^4^ km^2^).

Species	Moderately	highest	Total
*Trigonella arcuate*	65.49	29.09	448.78
*Tauscheria lasiocarpa*	255.46	155.32	1,802.66
*Anastatica hierochuntica*	155.32	46.51	448.45
*Trigonella arabica*	9.61	3.56	31.84
*Crocus alatavicus*	65.70	41.50	2,031.45
*Gagea filiformis*	112.52	60.49	557.14

Moderately suitable distribution means that the specific logistic threshold probability was between 0.4 and 0.6, and the highly suitable distribution was 0.6–1.

### Potential suitable habitat under different future climate change scenarios

**Figure 4:**
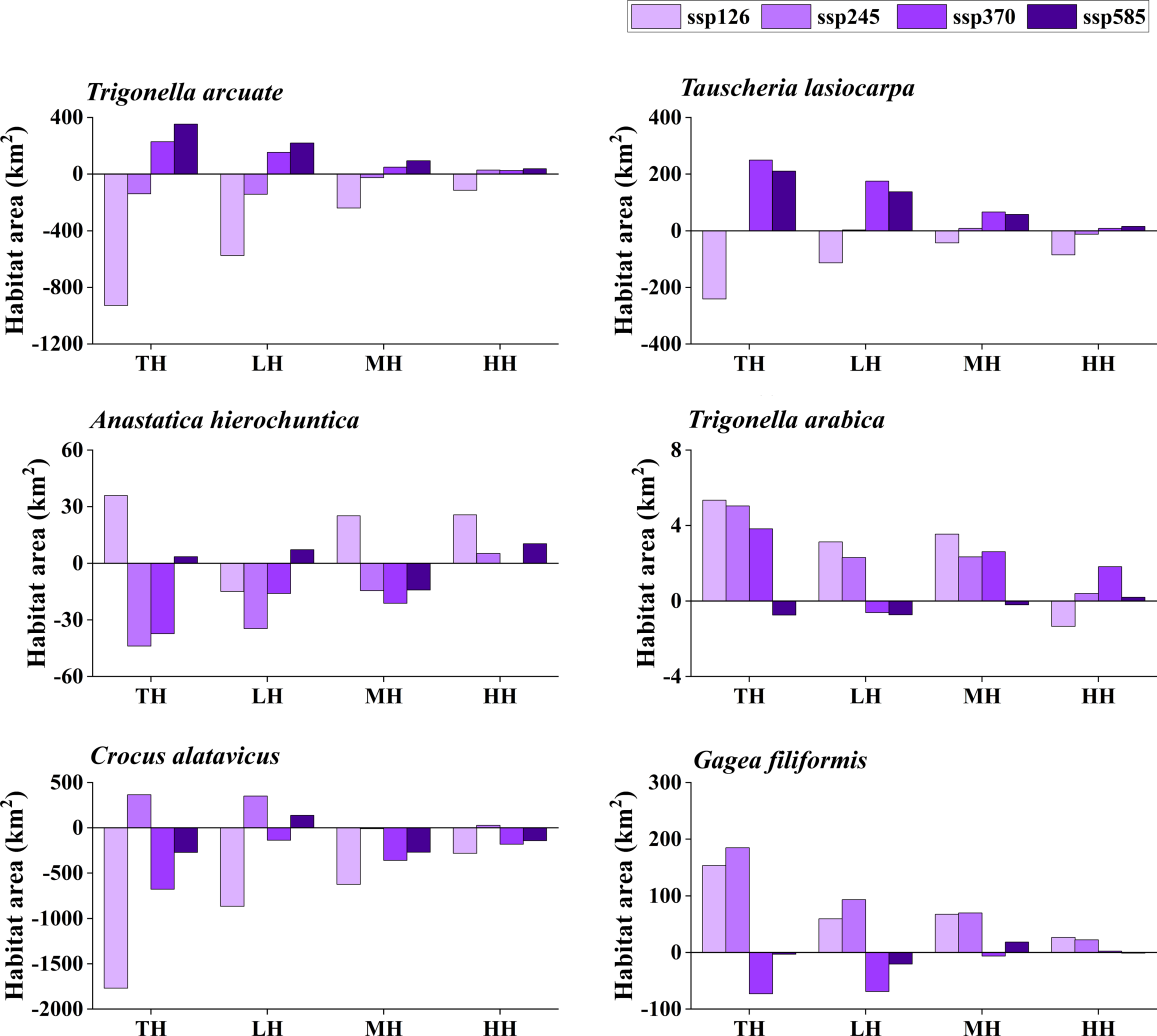
Potential suitable distribution habitat changes of six ephemeral plants under the current and 2021–2040 climate change scenarios (ssp 126, ssp 245, ssp 370, and ssp 585). TH, total suitable habitat; LH, lower suitable habitat; MH, moderately suitable habitat; HH, highly suitable habitat.

**Figure 5:**
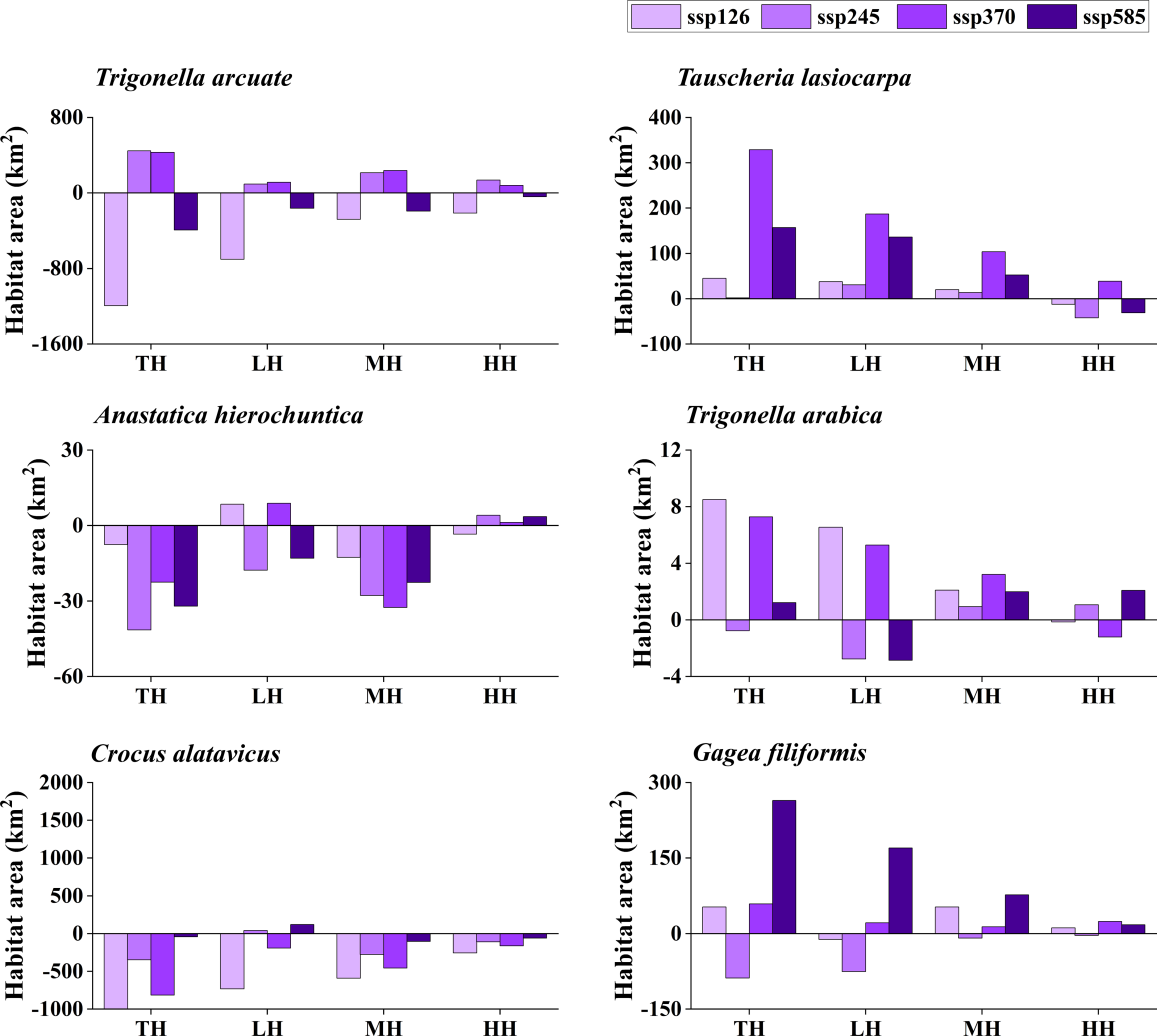
Potential suitable distribution habitat changes of six ephemeral plants under the current and 2041–2060 climate change scenarios (ssp 126, ssp 245, ssp 370, and ssp 585). TH, total suitable habitat; LH, lower suitable habitat; MH, moderately suitable habitat; HH, highly suitable habitat.

**Figure 6:**
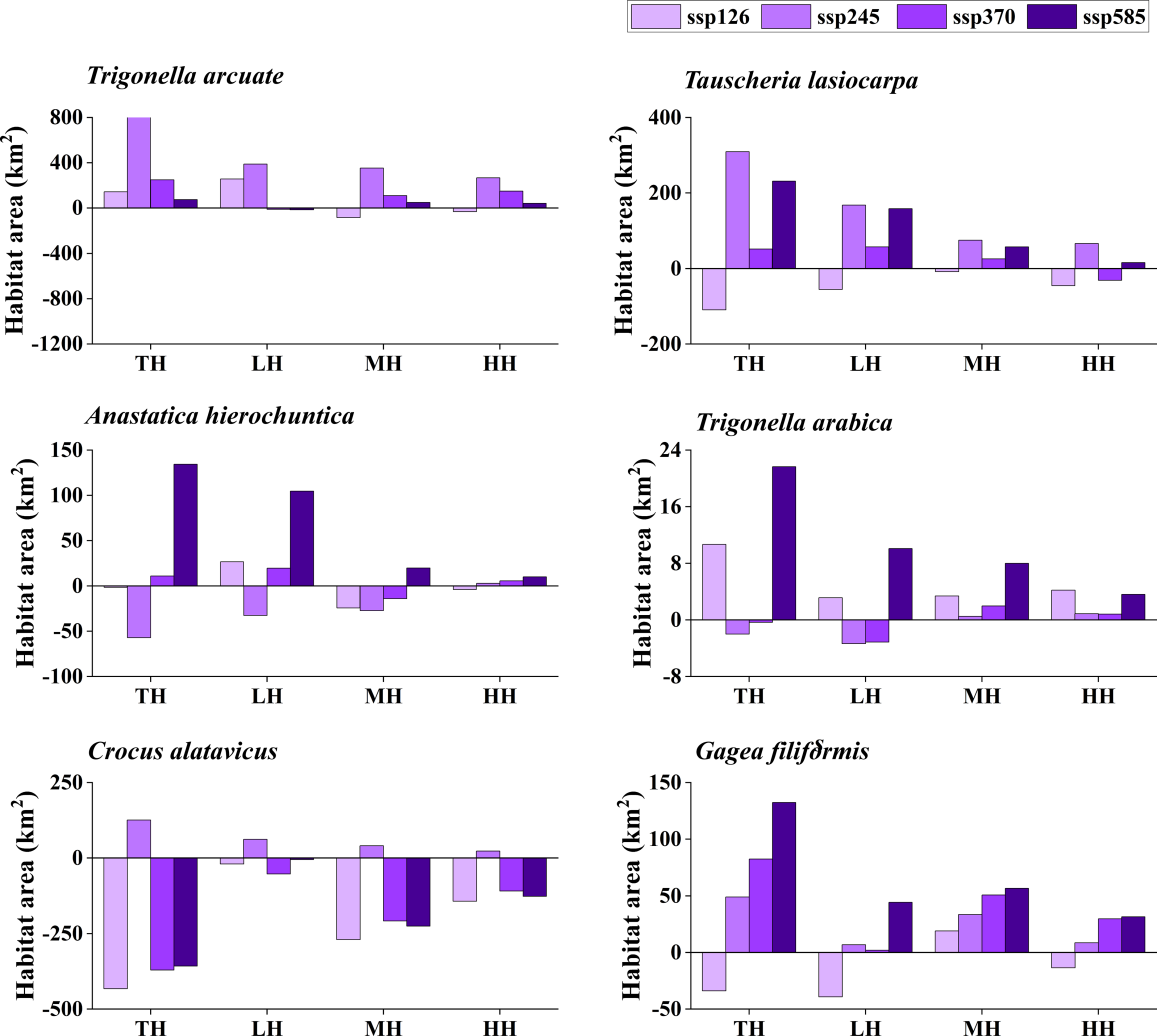
Potential suitable distribution habitat changes of six ephemeral plants under the current and 2061–2080 climate change scenarios (ssp 126, ssp 245, ssp 370, and ssp 585). TH, total suitable habitat; LH, lower suitable habitat; MH, moderately suitable habitat; HH, highly suitable habitat.

**Figure 7:**
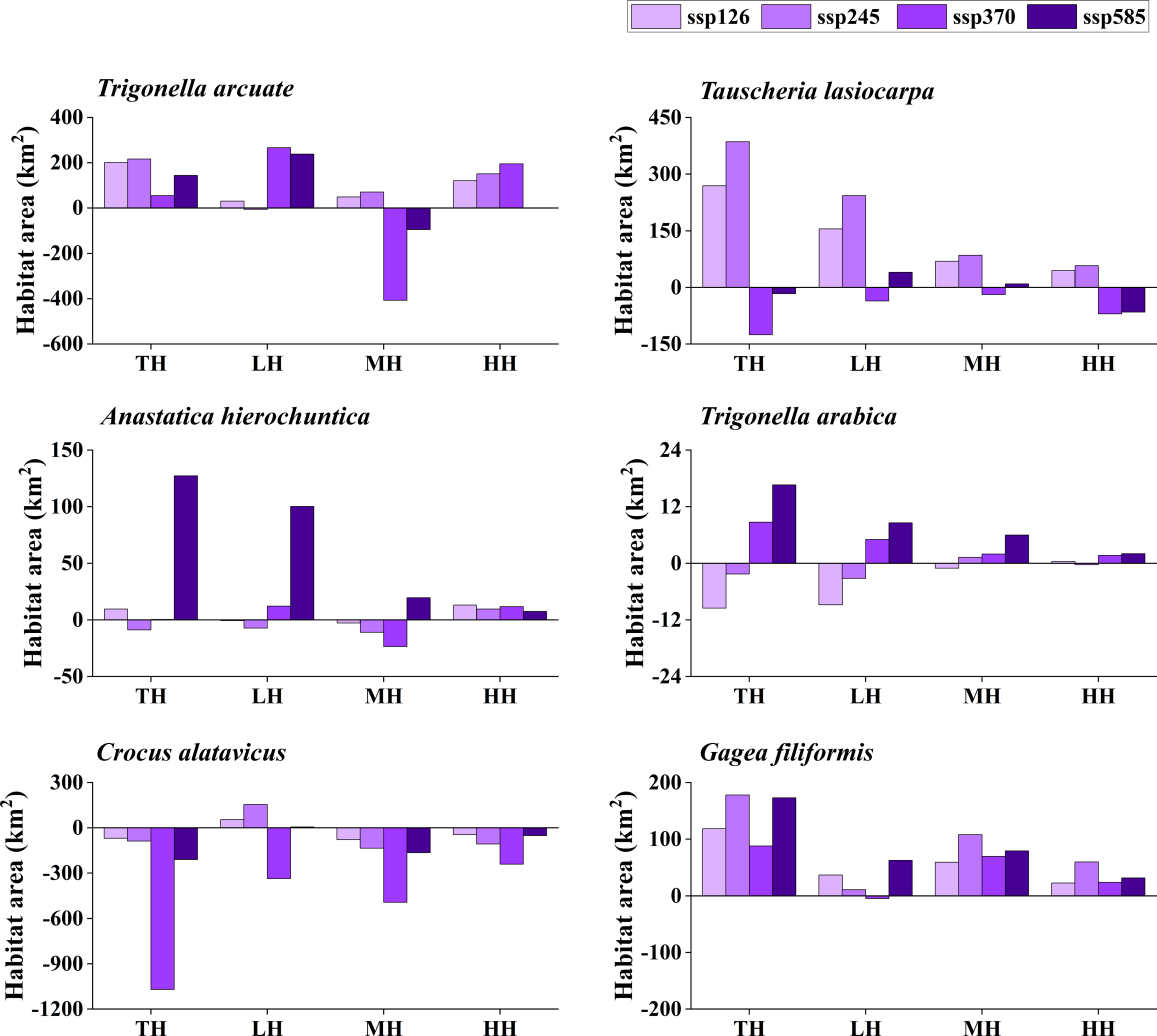
Potential suitable distribution habitat changes of six ephemeral plants under the current and 2081–2100 climate change scenarios (ssp 126, ssp 245, ssp 370, and ssp 585). TH, total suitable habitat; LH, lower suitable habitat; MH, moderately suitable habitat; HH, highly suitable habitat.

The potentially suitable distributions for three types of ephemeral plants were analyzed under 16 different future climatic scenarios (ssp 126, ssp 245, ssp 370, and ssp 585 in 2021–2040, 2041–2060, 2061–2080, and 2081–2100, respectively). The results indicate the changes in the potentially suitable area of six ephemeral plants with a significant difference (**Figures 4**, **5**, **6**, **7**).

In cold deserts, the total potential suitable area of *T. arcuate* increased under 11 climatic scenarios, and that of *T. lasiocarpa* increased under 12 climatic scenarios. The majority of climatic scenarios are under ssp 245, 370, and 585, which means that the distribution of cold desert ephemeral plants will increase under medium and high forces in future climate scenarios. For hot desert plants, the total potential suitable area of *A. hierochuntica* decreased under nine climatic scenarios (under most lower- and medium-force climate scenarios in 2021–2040 and 2041-2060) and increased in seven (under most high forces climate scenarios in 2061–2080 and 2081–2100). The total potential suitable distribution of *T. arabica* increased under 10 climatic scenarios, the majority are under high forces scenarios in 2061–2080 and 2081–2100. In deciduous forest ephemeral plants, the total potential suitable distribution of *C. alatavicus* decreased under 14 climatic scenarios. It indicates that the distribution area would be reduced with the increase in temperature. On the contrary, the total potential suitable distribution of *G. filiformis* increased under 12 climatic scenarios. The scenarios were under medium and high forces climate scenarios in 2061–2080 and 2081–2100.

## Discussion

### Major variables affecting the distribution of ephemeral plants

Ephemeral plants are a particular component of flora that take full advantage of light, water, and temperature to rapidly complete their life cycle in a very short time (Jeffrey, 1992). We use the MaxEnt model to identify the key factors to determine the distribution of ephemeral plants in three types of ecosystems under the current climate scenario. The results show that the significant variables affecting the potential suitable distribution in three ecosystems were inconsistent with our hypothesis.

#### Cold deserts

In the cold desert, the distribution patterns of ephemeral plants were thought to be regulated by rainfall and snowmelt, especially the spring rainfall. Duan et al. (2017) observed that increased precipitation has led to an increase in the distribution area of ephemeral plants in the Gurbantunggut Desert in China over the past 30 years. In addition, Wang et al. (2006) also considered surface soil moisture as the essential factor affecting the distribution of ephemeral plants in early spring. However, in our result, precipitation and soil moisture were important factors in determining ephemeral plants in cold deserts. Soil pH was still the main factor in determining the distribution of two ephemeral plants (**Table 4**). One reason is related to the historical process of evolution and formation (Peng et al., 2022). The ephemeral plants in cold deserts are drought escape species that formed after the ebbing of the Tethys Sea, and their pH-adapted range is not too high nor too low, which therefore limits their area of distribution and evolutionary traits (Peng et al., 2022). Meanwhile, there was lower precipitation in summer and autumn and a large amount of rainfall in winter and spring. The cold desert ephemeral plants could use snowmelt and rain in early spring to grow and shorten the life cycle to complete the reproductive growth quickly. Therefore, precipitation was not the most critical limiting factor for the distribution of these cold desert ephemeral plants.

Our research also found that the annual mean temperature has the second contribution to the distribution of cold desert plants (**Table 4**). The distribution of ephemeral plants in the cold desert has unique regional and seasonal characteristics. The stable snowfall covered the ground in winter; with temperature rises, snowmelt provides a suitable environment for seed germination and seedling growth of ephemeral plants (Fan et al., 2012). For seed germination, it is the beginning of plant life history. This process was related to the seedling and growth of ephemeral plants and the distribution and expansion of populations. For example, in Gurbantunggut Desert, China, the temperature is extremely low in winter (almost -20°C), but in early spring the temperature can rapidly rise to 5-10°C. This alternating temperature change is the most suitable environmental condition to break the ephemeral plant’s seed dormancy. For plant growth and development, increased temperature could accelerate plant metabolism and stimulates plant growth and development. Still, it reduces soil moisture and limits plant biomass accumulation. At the same time, the significant temperature differences between seasons keep the annual mean temperature within a small range. Therefore, the annual mean temperature was a secondary factor in determining the distribution of ephemeral plants in cold desert ecosystems.

#### Hot deserts

Hot desert are often distributed between 20° to 33° north and south latitudes, and climates are typically subtropics (Ezcurra et al., 2014). In these regions, descending air and high pressure aloft create the intense sunshine and arid conditions whole year. Plants have evolved multiple mechanisms to adapt to extreme hot and drought climates (Alberto, 1999).

We found that the precipitation of the driest month contributed the largest to *A. hierochuntica* and *T. arabica* distribution in hot deserts (**Table 5**), and the precipitation of the driest month ranges close to 0 mm. In hot deserts, maximum air temperatures could be over 40°C in summer and soar to over 45°C. Such dry and hot climatic conditions allow for the presence of a small number of plants with remarkable adaptive capabilities (Robichaux, 1999). Studies found that many ephemeral plant seeds may remain viable for decades in dry soil; when sufficient rainfall and soil moisture conditions come, they can germinate, grow, and reproduce (Whitford & Duval, 2020). Thus, those plants have a short life cycle, use seed dormancy to escape the resistance, and can still distribute in the hot region.

However, hot desert plants have a more specific adaptation strategy to cope with extreme drought and high-temperature climates. These resurrection plants (*A. hierochuntica*) confront extreme desiccation by shifting into a dormant state (Moore et al., 2007). Even when their body loses water content by nearly 95%, they can still return to total activity upon rehydration (Peter, 2000). The resurrection plants can curl their branches so that the seeds could be protected in good conditions from the hostile environment; therefore, the species can survive for years in hot deserts (Bernacchia et al., 1996). In the hot desert, such as the central Sahara Desert, the annual total precipitation is less than 1 mm. However, the Sahara’s precipitation is unpredictable—for example, the low-pressure near the equator brings short, irregular rainfall to the Sahara (Celestin et al., 2021). The resurrection plant could wake up and uncurl the branches in a few minutes. Thus, new shoots will flourish in a very short time after releasing the seeds (Moore et al., 2007). When the dry season comes again, they will return to a dormant state until the next precipitation event (Moore et al., 2007). Thus, unique strategies can help resurrection plants live in arid and hot environments.

#### Deciduous forest

In deciduous forests, ephemeral plants use the high-light period available in early spring. They are primarily perennials that usually complete their life history in early spring before the tree canopy cover. Their life cycle was also concise; within 2 months, they can complete the aboveground (including fruit production) growth, and then they senesce and enter dormancy with underground organs, but the dormancy was not very deep (Mckenna & Houle, 1999). Differentiation can occur in the bud of the underground organ during summer.

Studies believe that temperature was a pivotal factor affecting the growth of ephemeral plants in early spring. Low soil temperatures can break the dormancy and promote biomass accumulation in plants—for instance, the final biomass of *C. vernus* at 12°C was higher than at 18°C (Badri et al., 2007); similar results also have been observed in *Allium tricoccum* (Nault & Gagnon, 1993), *E. americanum* (Lapointe & Lerat, 2006), and many spring bulbous species (Hertogh & Nard, 1993; Badri et al., 2007). The studies mentioned earlier support our results that temperature was the main factor in determining the distribution of *C. alatavicus* and *G. filiformis* (**Table 6**).

The lower temperature could put off the tree’s phenology and delay the leaf expansion. Therefore, the ephemeral plants were unrestricted by light resources and had a more extended period for nutritional and reproductive growth. Thus, the low temperature favored ephemeral plant distribution in deciduous forests.

### Response of ephemeral plants to climate change

Compared to the potential distribution change between current and future scenarios modeled by the MaxEnt model, the results show that the distributions of ephemeral plants in the three ecosystems will be changing under most future climate scenarios (SSP126, SSP245, SSP370, and SSP585 in 2021–2040, 2041–2060, 2061–2080, and 2081–2100) (*T. arcuate* 11 to 16 and *T. lasiocarpa* 12 to 16).

Studies found that the global arid regions have been expanding significantly due to precipitation decreases and temperature increases over the past 60 years (Feng & Fu, 2013; Huang et al., 2015). The trends will continue in the future, especially in mid-latitude arid and semi-arid regions. Lower precipitation and higher temperature may offer more suitable areas for cold desert plants to survive. However, Chen (2021) found that the arid zones may shrink by the end of the 21st century under RCP8.5 (the highest concentration of greenhouse gas emissions in the future) and RCP4.5 (moderate concentration of greenhouse gas emissions in the future) scenarios, such as in northern China and India. The results were consistent with the finding that the potential distribution area for cold desert ephemeral plants would decrease under most future climate scenarios (**Figures 4**–**7**). Although shrinkage of the arid area may occur in the cold desert regions of Central Asia, the extent is much lower than the expansion (Chen, 2021). The total aridity region still shows an expanding trend and will likely expand by approximately 10% by the end of this century. It indicates that global climate change could provide more areas for ephemeral plants to survive in the cold desert.

Several studies show that hot desert regions may expand under future climate scenarios, such as in North Africa and Saudi Arabia (Vale & Brito, 2015; Chen, 2021). Our study’s potential distribution area of *A. hierochuntica* decreases in more than half of future climate scenarios (**Figures 4**–**7**). It was inconsistent with our hypothesis that the ephemeral plant in hot desert distribution will be expanding in the future. One possibility was that the plants were distributed in North Africa, Egypt, Morocco, Libya, *etc*. The annual mean precipitation was virtually zero in Libya, Egypt, and Sudan. Still, the Sahara’s rainfall is unreliable and erratic (Vale & Brito, 2015). This uncertainty leads to the prediction results having significant differences. The potential distribution was decreased under nearly half of the future climate scenarios of *T. arabica* and increased by roughly half of those of *A. hierochuntica*. It suggests that climate change leads to arid area expansion in the future, which may not significantly affect the distribution of hot desert ephemeral plants.

The deciduous forest ephemeral plant potential distribution area of *C. alatavicus* was decreased under the majority of future climatic scenarios (**Figures 4**–**7**), which is consistent with our hypothesis that the distribution of deciduous forest ephemeral plant would be reduced in the future. Zhang and Hu (2009) believed that global warming negatively impacts forest ephemeral plants. If the temperature rises by 1 to 2°C, the potential distribution of early ephemeral plants would be reduced or more fragmented, which may cause some species’ suitable habitats to become reduced or even extinct. Therefore, with the temperature increase in future climate scenarios, the suitable habitats of *C. alatavicus* would be further compressed.

However, our study found that the potential distribution of *G. filiformis* was increased in multiple future climate scenarios, which is inconsistent with our hypothesis. The results are due to the main limiting factors between *C. alatavicus* and *G. filiformis* being different. The annual mean temperature had the highest contribution for *C. alatavicus*; the pH had the highest for *G. filiformis*. The difference in determining factors may indicate the adaptation strategies in species. The potential distribution change was variance in ephemeral plants under the future climate change scenarios: ephemeral plant distribution in cold deserts were increased under the majority of future climate scenarios, but in hot deserts, the distribution was relatively decreased; but in deciduous forest widely-distributed species would decrease, narrow range species would increase. Thus, ephemeral plants with greater adaptive capacity are likely to occupy a wide range of niches, while the niches of those with lower adaptive capacity will shrink further under climate change. This information may be useful for formulating relevant policies to prevent managed species with an expanding potential distribution from becoming invasive species, and for shrinking species, we should minimize damage and protect them from species and in avoid further reductions in species range and their distribution in the future.

### Uncertainty

In our research, only five soil conditions were input to the MaxEnt model; meanwhile, we also hypothesized that the soil conditions would remain unchanged in the future. Changes in soil conditions should be considered to better understand the distribution of ephemeral plants. Secondly, desert ephemeral plants had only two or three months in early spring to complete the life cycle. However, ephemeral plants may occur in suitable (light, precipitation, temperature) conditions; even in autumn, the seasonal change was not considered. These may explain why the ephemeral plant often occurs in summer and autumn.

## Conclusions

This study has identified six ephemeral plants in different habitats, belonging to the cold desert, hot desert, and deciduous forest. The average AUC value of each species was higher than 0.95, indicating that the MaxEnt model for each species was excellent. Cold desert and deciduous forest ephemeral plant distributions were mainly determined by soil pH and annual mean temperature, and the main factor in hot deserts was precipitation in the driest month. The potential suitable distribution of ephemeral plants in the cold desert was increased under one-third of future climate scenarios. However, in the hot desert, it decreased nearly half of the future climate scenarios of *T. arabica*, and roughly half increased of *A. hierochuntica*. In deciduous forest ephemeral plants, *C. alatavicus* decreased in the near absolute majority of future climate scenarios, and the *G. filiformis* increased in 75% of climate scenarios. These results indicate that the potential suitable distribution of ephemeral plants in these different ecosystems was closely related to the adaptation strategies of each species. These results contribute to a comprehensive understanding of the potential distribution pattern and the suitable habitat distribution of some ephemeral plants in arid and semi-arid ecosystems.

## Data availability statement

The original contributions presented in the study are included in the article/**Supplementary Material**. Further inquiries can be directed to the corresponding authors.

## Author contributions

ZL, LH, ZH, CY, and ZY conceived and designed the study. ZL, CY, KK, and DT analyzed the data. ZL, LH, and YC wrote the manuscript. All authors contributed to the article and approved the submitted version.

## Funding

This research was supported by the financial support of the National Natural Science Foundation of China (32171513, 32160526, 31971428, and U2003214), the Youth Innovation Promotion Association of the Chinese Academy of Sciences (2018477), and the Chinese Academy of Sciences Pioneer A Project (XDA2005020402).

## Acknowledgments

We gratefully acknowledge the support of the National Natural Science Foundation of China, the Youth Innovation Promotion Association of the Chinese Academy of Sciences, and the Chinese Academy of Sciences Pioneer A Project. We thank Wen Xiaohu and Lu Yuting for online data collection.

## Conflict of interest

The authors declare that the research was conducted in the absence of any commercial or financial relationships that could be construed as a potential conflict of interest.

## Publisher’s note

All claims expressed in this article are solely those of the authors and do not necessarily represent those of their affiliated organizations, or those of the publisher, the editors and the reviewers. Any product that may be evaluated in this article, or claim that may be made by its manufacturer, is not guaranteed or endorsed by the publisher.
