# Supplementary material for: Potential distribution of three types of ephemeral plants under climate changes
Source: Front Plant Sci. 2022 Nov 23;13:1035684. doi: 10.3389/fpls.2022.1035684 (PMC9728545; doi:10.3389/fpls.2022.1035684)
Supplement: Supplementary file 1 [file DataSheet_1.pdf]

Figure supply:

Figure 1s. Response curves for the eight main environmental variables affecting the potential suitable area of *Trigonella arcuate*.

Figure 2s. Response curves for the eight main environmental variables affecting the potential suitable area of *Tauscheria lasiocarpa*.

Figure 3s. Response curves for the eight main environmental variables affecting the potential suitable area of *Anastatica hierochuntica*.

Figure 4s. Response curves for the eight main environmental variables affecting the potential suitable area of *Trigonella arabica*.

Figure 5s. Response curves for the eight main environmental variables affecting the potential suitable area of *Gagea filiformis*.

Figure 6s. Response curves for the eight main environmental variables affecting the potential suitable area of *Crocus alatavicus*.

Figure 7s. Potential distribution of *Trigonella arcuate* in 2021-2040.

Figure 8s. Potential distribution of *Trigonella arcuate* in 2041-2060.

Figure 9s. Potential distribution of *Trigonella arcuate* in 2061-2080.

Figure 10s. Potential distribution of *Trigonella arcuate* in 2081-2100.

Figure 11s. Potential distribution of *Tauscheria lasiocarpa* in 2021-2040.

Figure 12s. Potential distribution of *Tauscheria lasiocarpa* in 2041-2060.

Figure 13s. Potential distribution of *Tauscheria lasiocarpa* in 2061-2080.

Figure 14s. Potential distribution of *Tauscheria lasiocarpa* in 2081-2100.

Figure 15s. Potential distribution of *Anastatica hierochuntica* in 2021-2040.

Figure 16s. Potential distribution of *Anastatica hierochuntica* in 2041-2060.

Figure 17s. Potential distribution of *Anastatica hierochuntica* in 2061-2080.

Figure 18s. Potential distribution of *Anastatica hierochuntica* in 2081-2100.

Figure 19s. Potential distribution of *Trigonella arabica* in 2021-2040.

Figure 20s. Potential distribution of *Trigonella arabica* in 2041-2060.

Figure 21s. Potential distribution of *Trigonella arabica* in 2061-2080.

Figure 22s. Potential distribution of *Trigonella arabica* in 2081-2100.

Figure 23s. Potential distribution of *Gagea filiformis* in 2021-2040.

Figure 24s. Potential distribution of *Gagea filiformis* in 2041-2060.

Figure 25s. Potential distribution of *Gagea filiformis* in 2061-2080.

Figure 26s. Potential distribution of *Gagea filiformis* in 2081-2100.

Figure 27s. Potential distribution of *Crocus alatavicus* in 2021-2040.

Figure 28s. Potential distribution of *Crocus alatavicus* in 2041-2060.

Figure 29s. Potential distribution of *Crocus alatavicus* in 2061-2080.

Figure 30s. Potential distribution of *Crocus alatavicus* in 2081-2100.

Cold desert

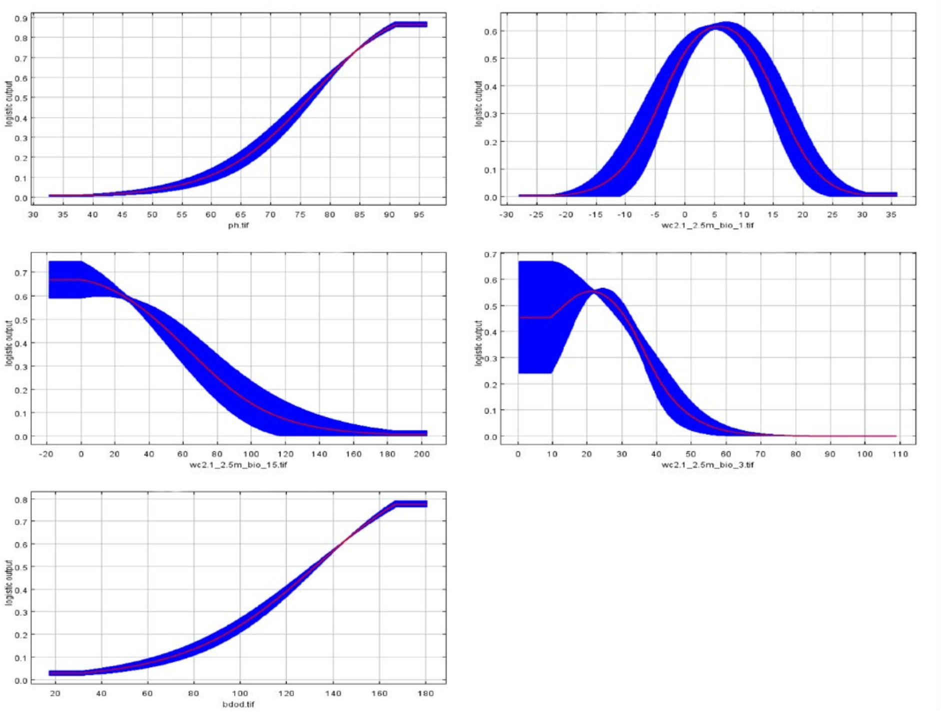

Figure 1s. Response curves for the eight main environmental variables affecting the potential suitable area of *Trigonella arcuate*.

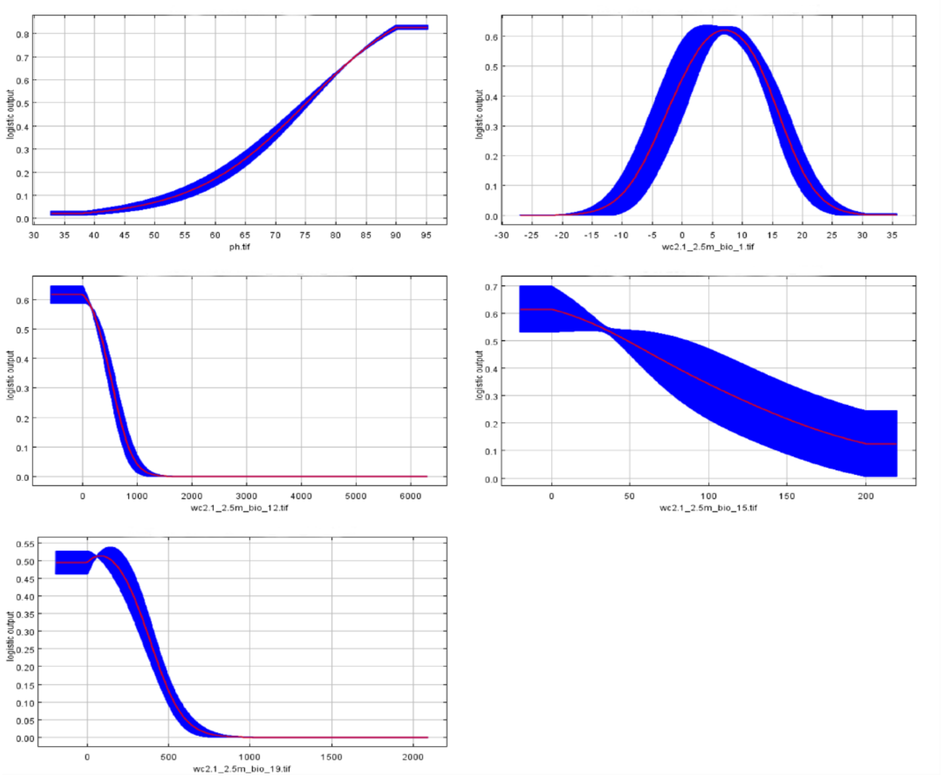

Figure 2s. Response curves for the eight main environmental variables affecting the potential suitable area of *Tauscheria lasiocarpa*.

Hot desert

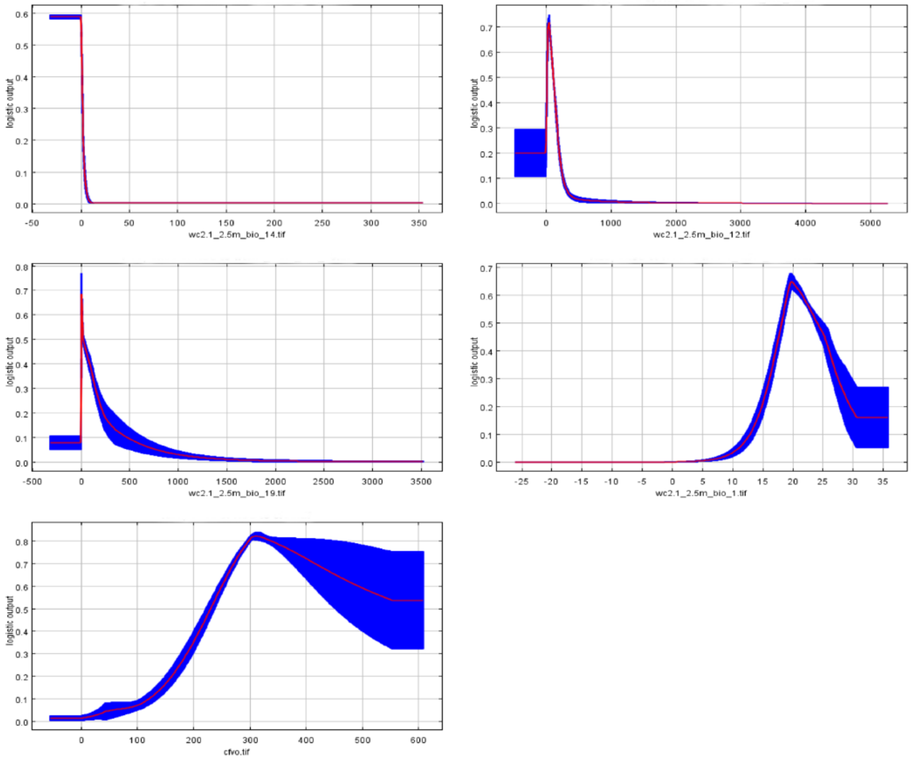

Figure 3s. Response curves for the eight main environmental variables affecting the potential suitable area of *Anastatica hierochuntica*.

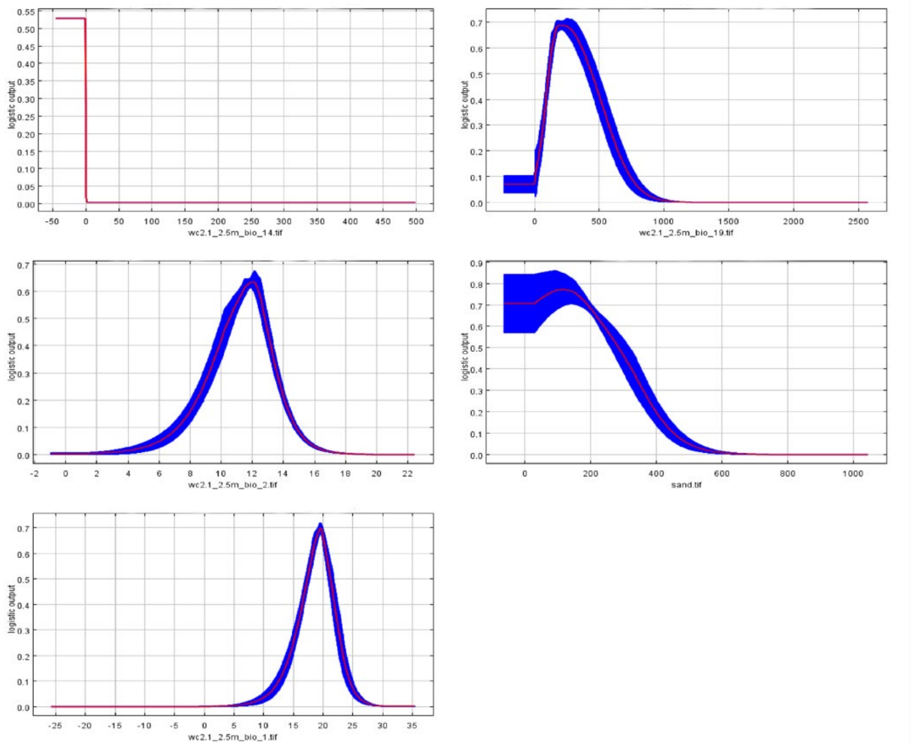

Figure 4s. Response curves for the eight main environmental variables affecting the potential suitable area of *Trigonella arabica*.

Deciduous Forest

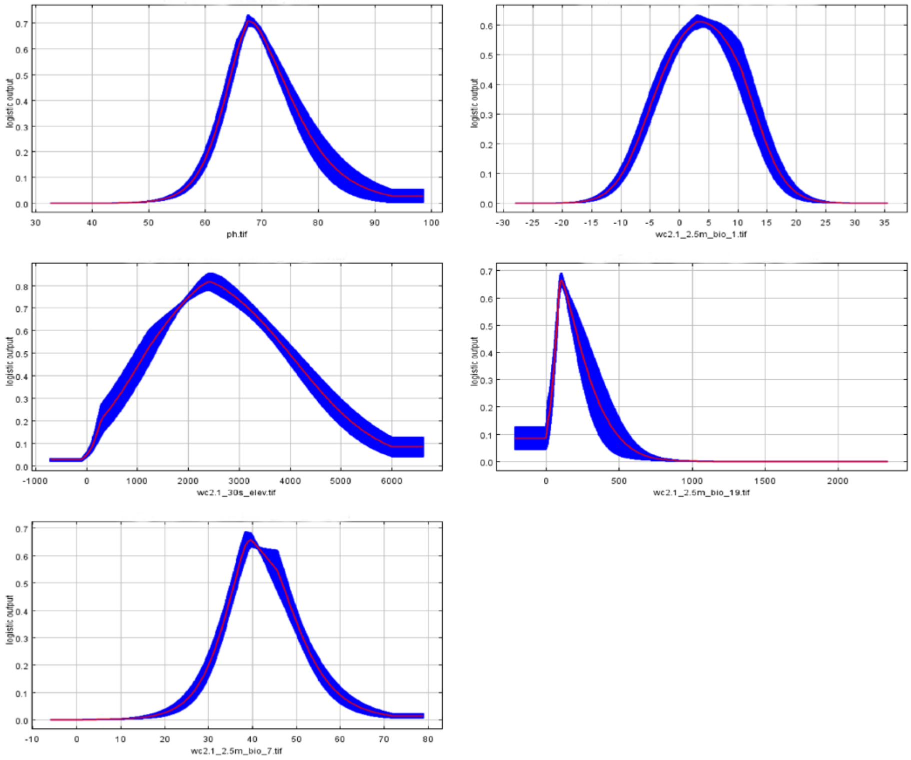

Figure 5s. Response curves for the eight main environmental variables affecting the potential suitable area of *Gagea filiformis*.

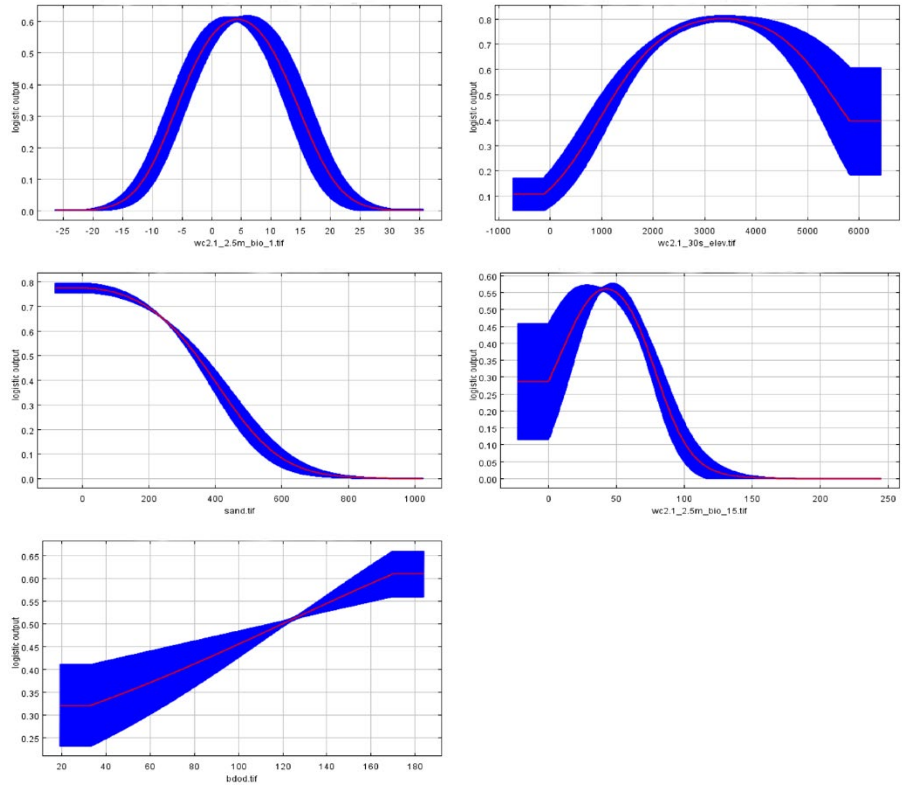

Figure 6s. Response curves for the eight main environmental variables affecting the potential suitable area of *Crocus alatavicus*.

Cold desert

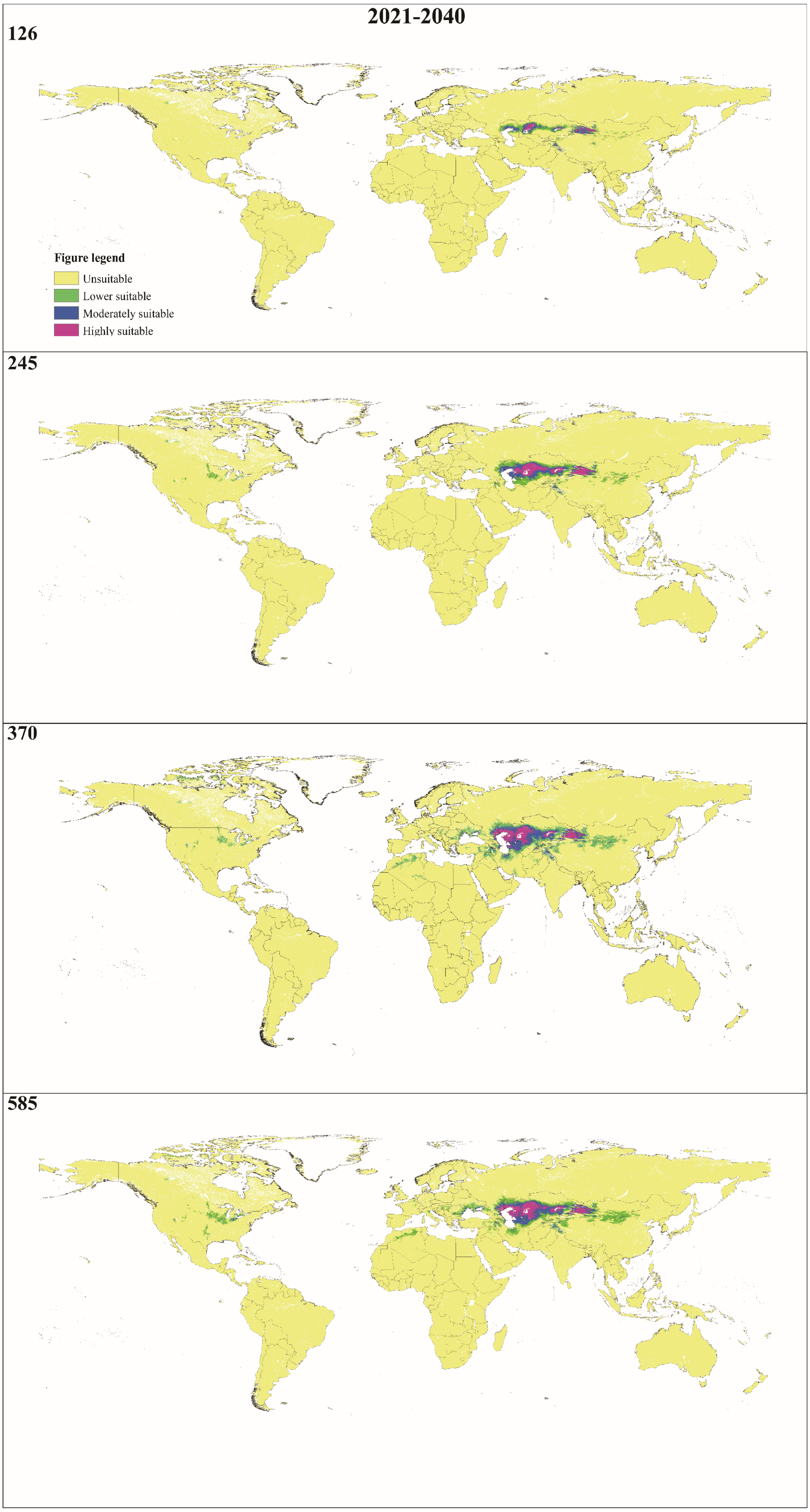

Figure 7s. Potential distribution of *Trigonella arcuate* in 2021-2040.

2041-2060

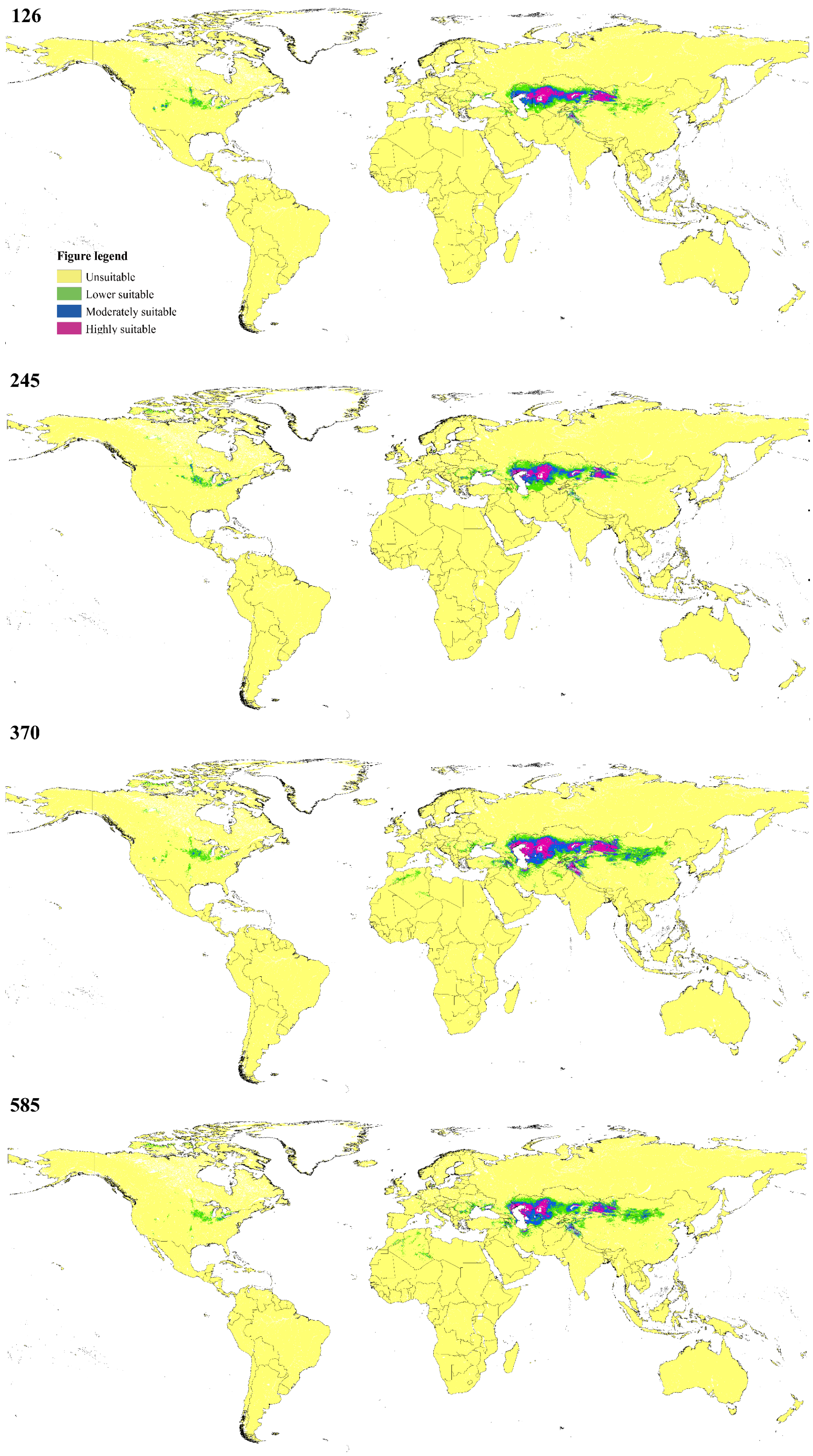

Figure 8s. Potential distribution of *Trigonella arcuate* in 2041-2060.

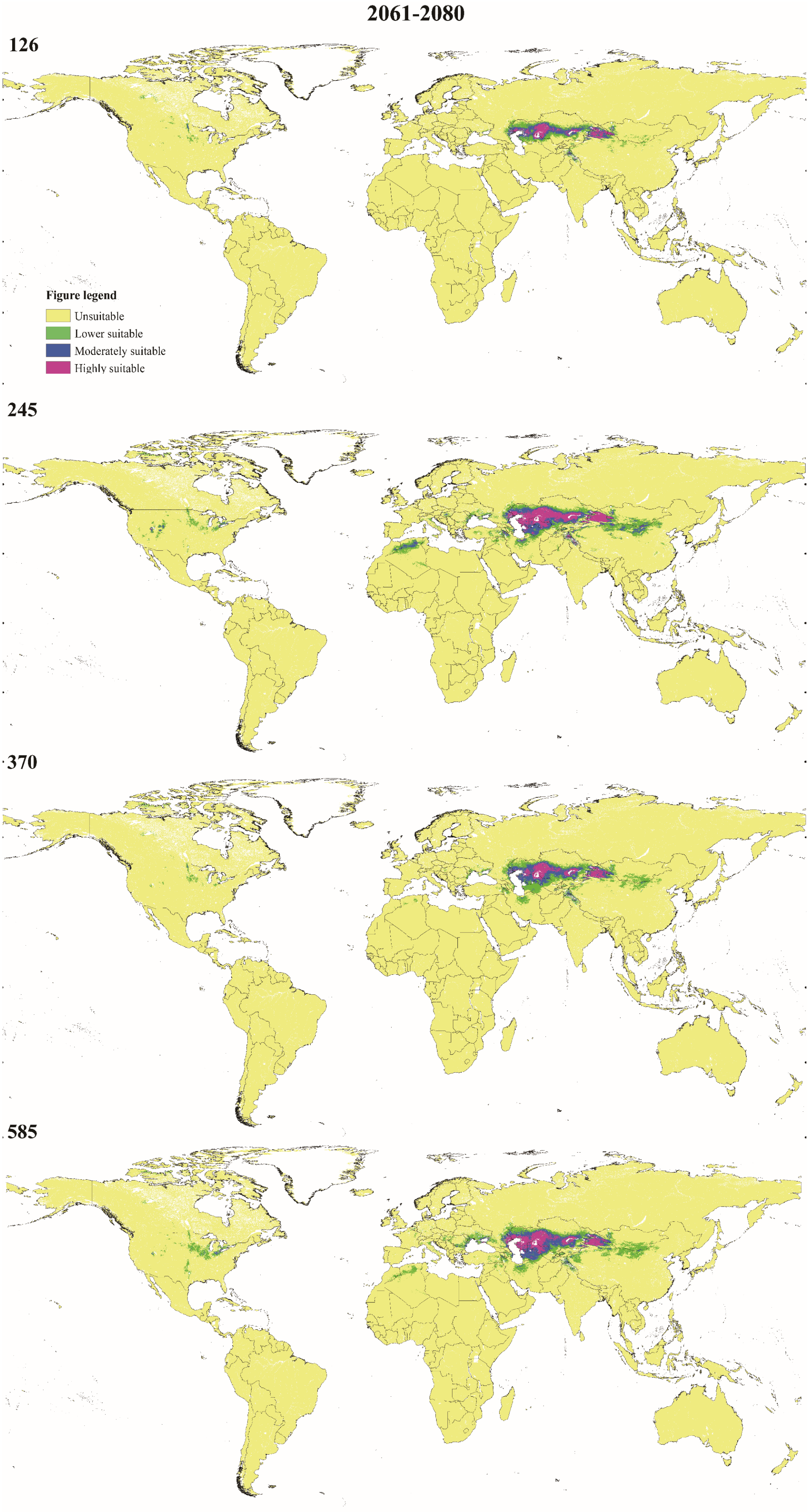

Figure 9s. Potential distribution of *Trigonella arcuate* in 2061-2080.

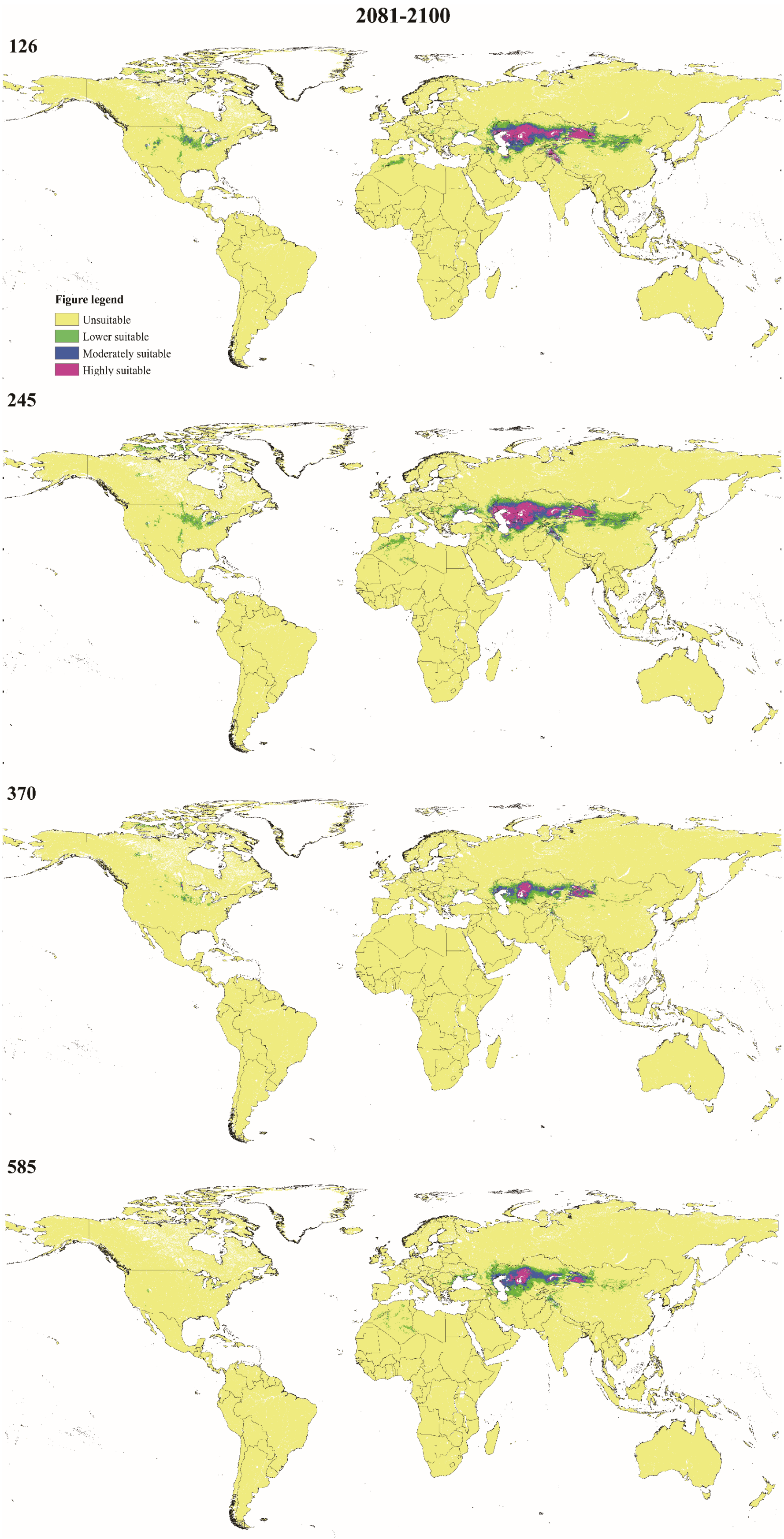

Figure 10s. Potential distribution of *Trigonella arcuate* in 2081-2100.

2021-2040

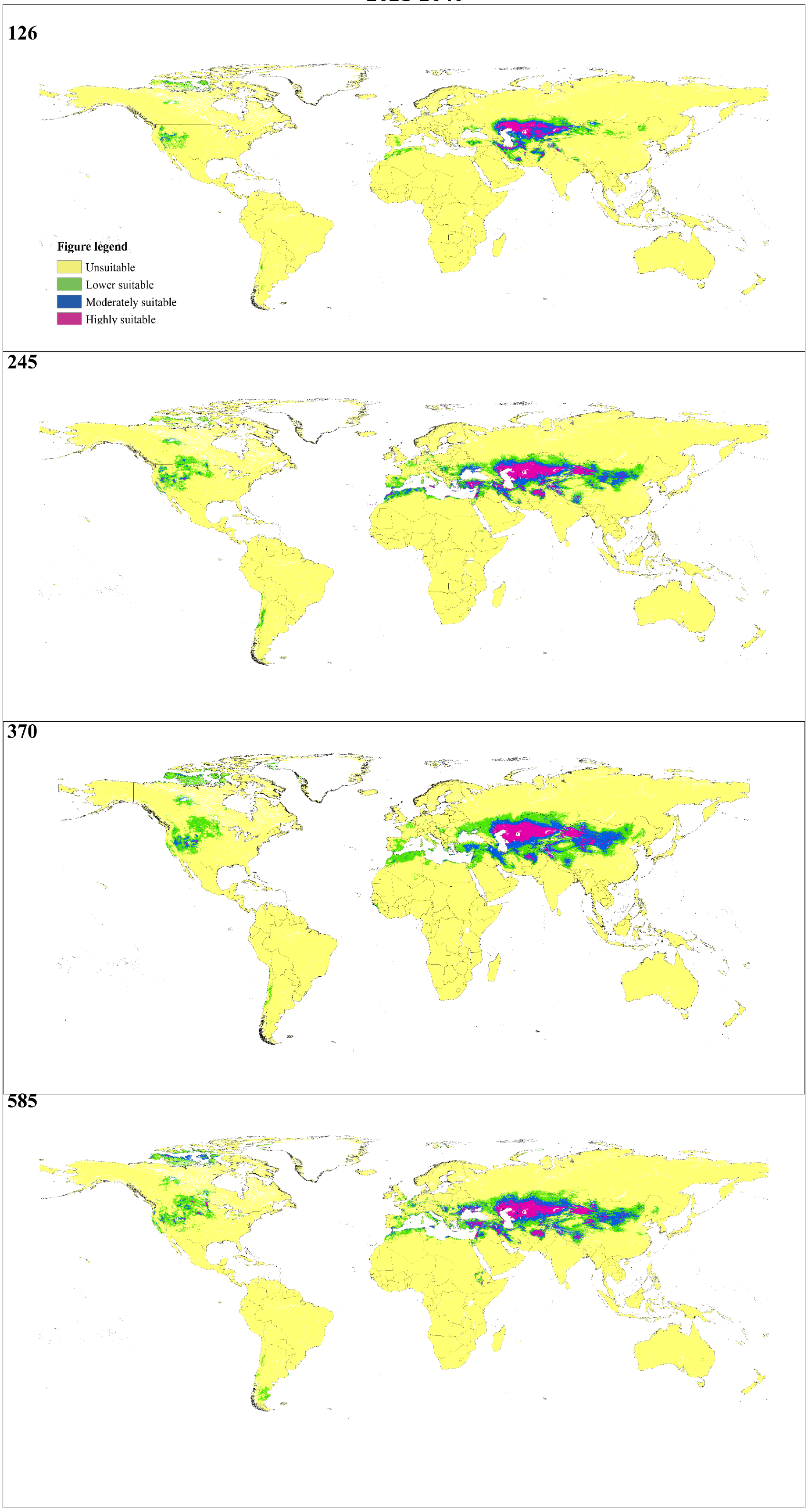

Figure 11s. Potential distribution of *Tauscheria lasiocarpa* in 2021-2040.

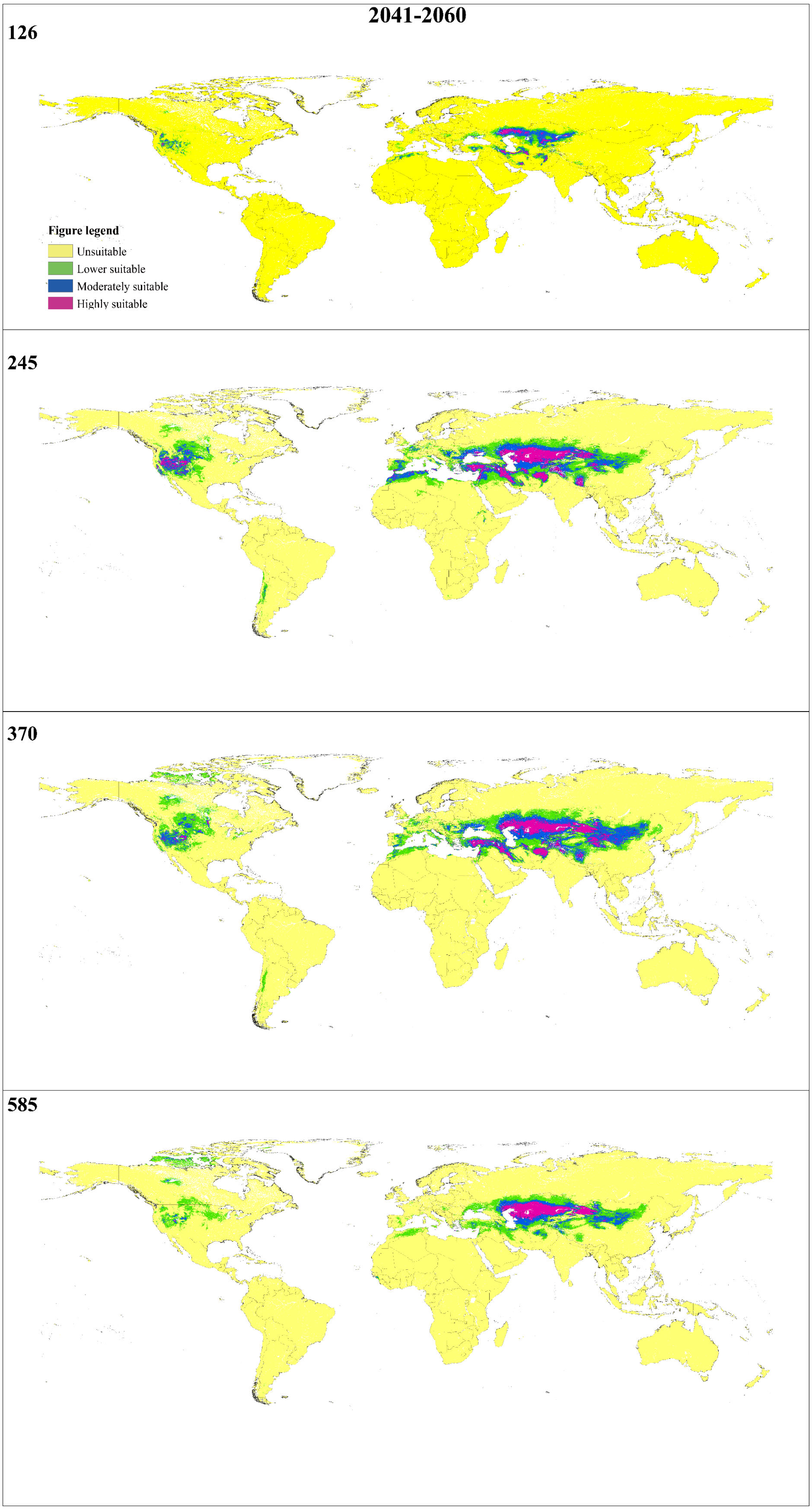

Figure 12s. Potential distribution of *Tauscheria lasiocarpa* in 2041-2060.

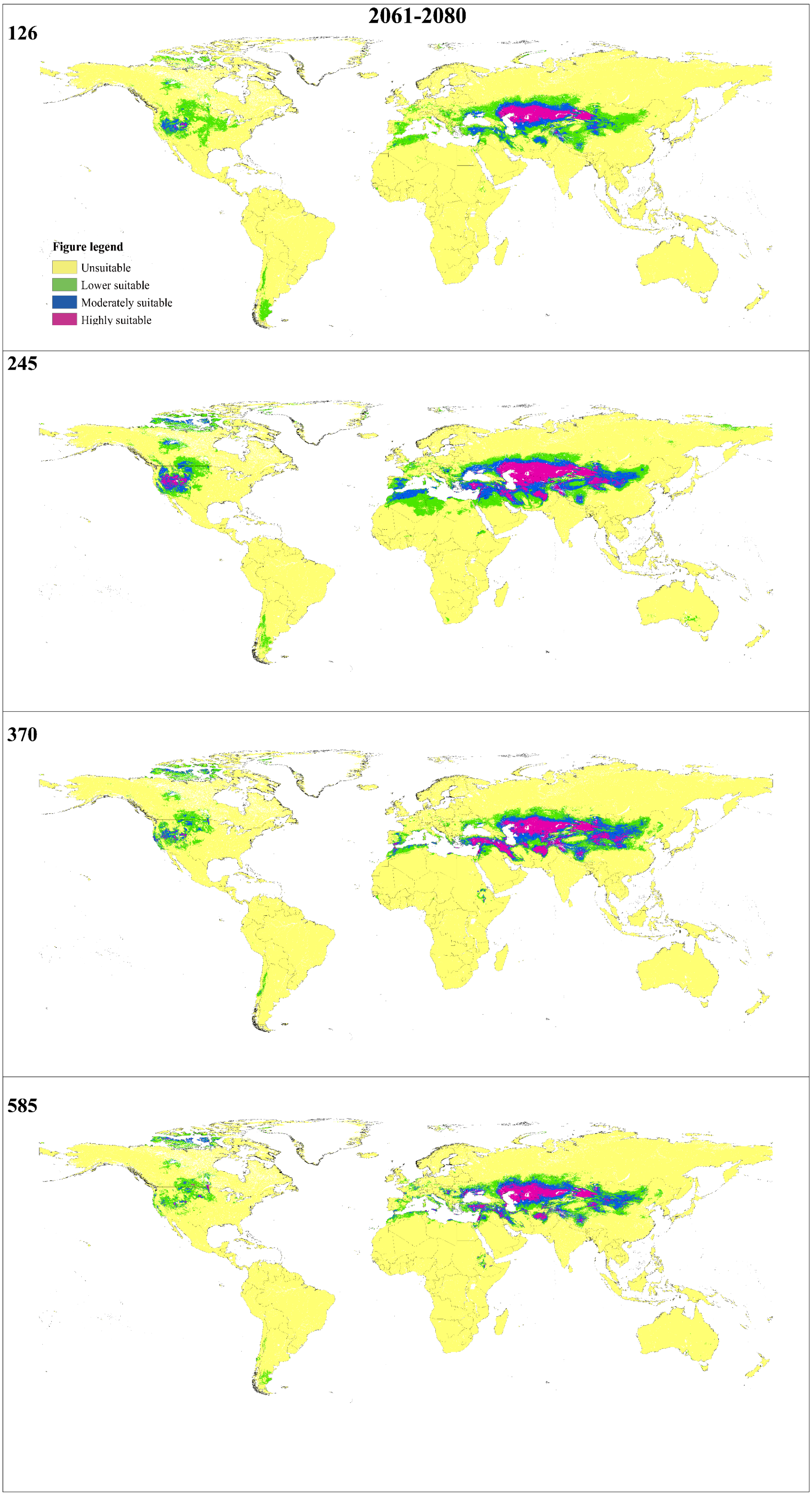

Figure 13s. Potential distribution of *Tauscheria lasiocarpa* in 2061-2080.

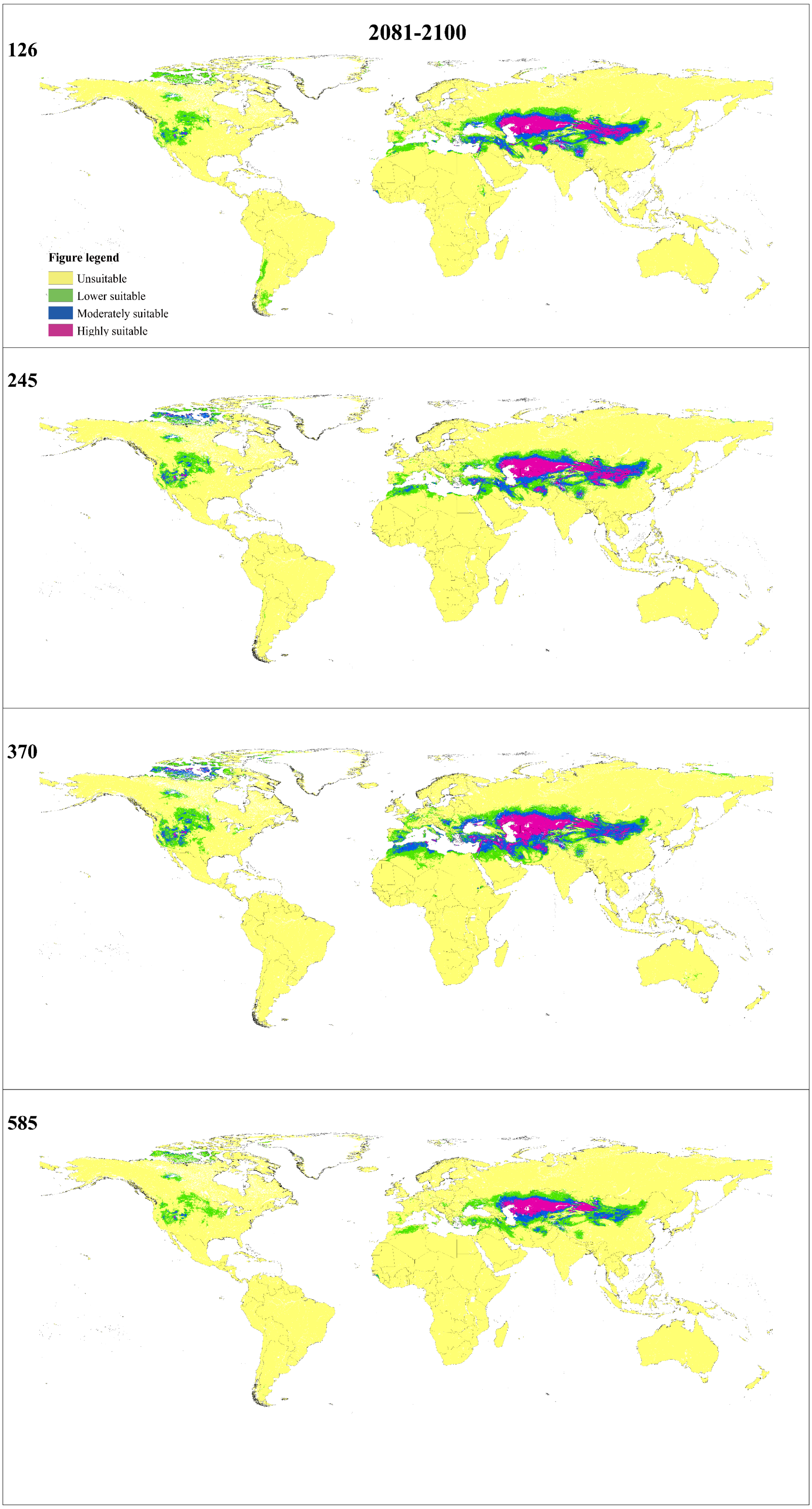

Figure 14s. Potential distribution of *Tauscheria lasiocarpa* in 2081-2100.

Hot desert

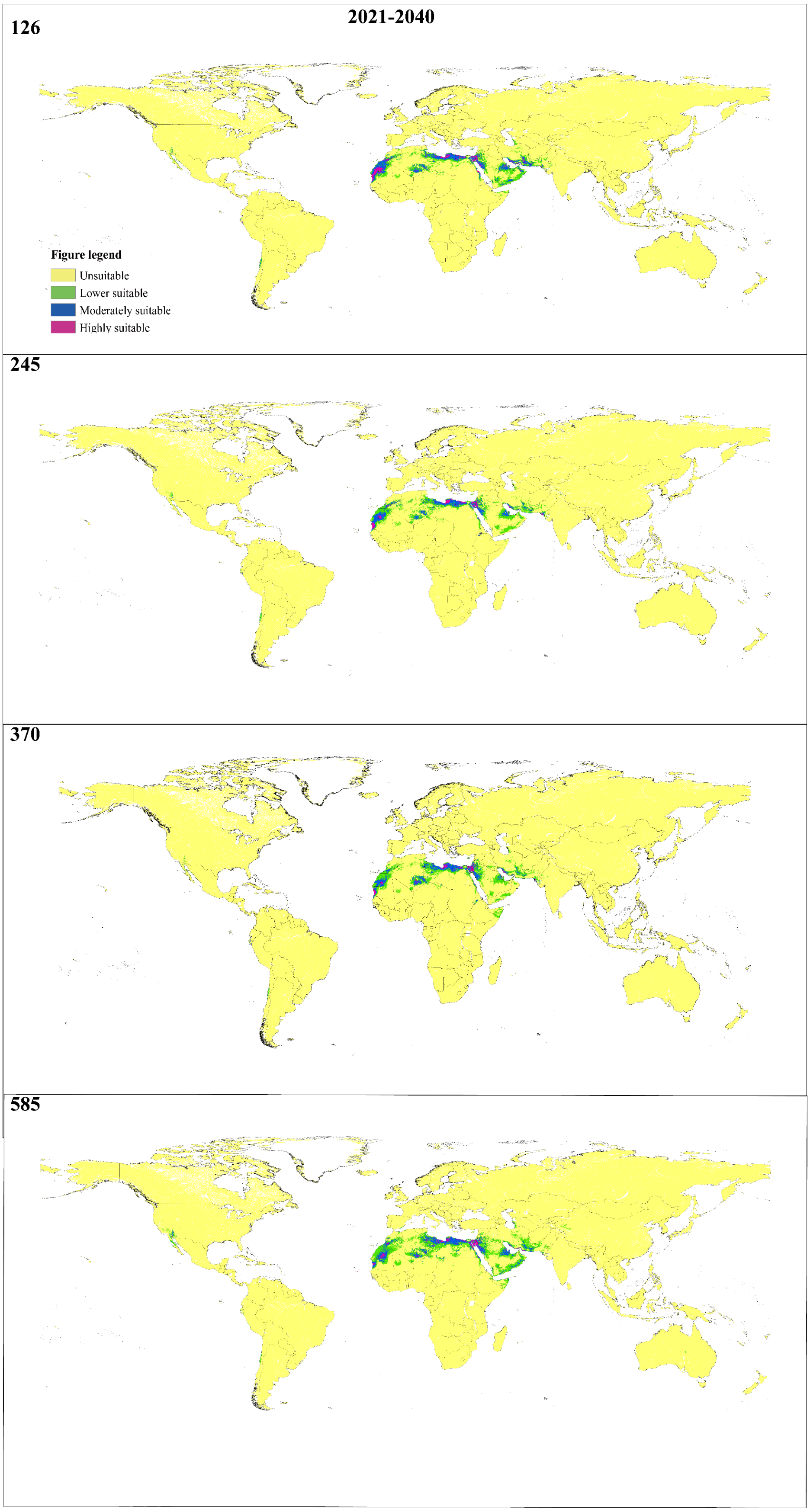

Figure 15s. Potential distribution of *Anastatica hierochuntica* in 2021-2040.

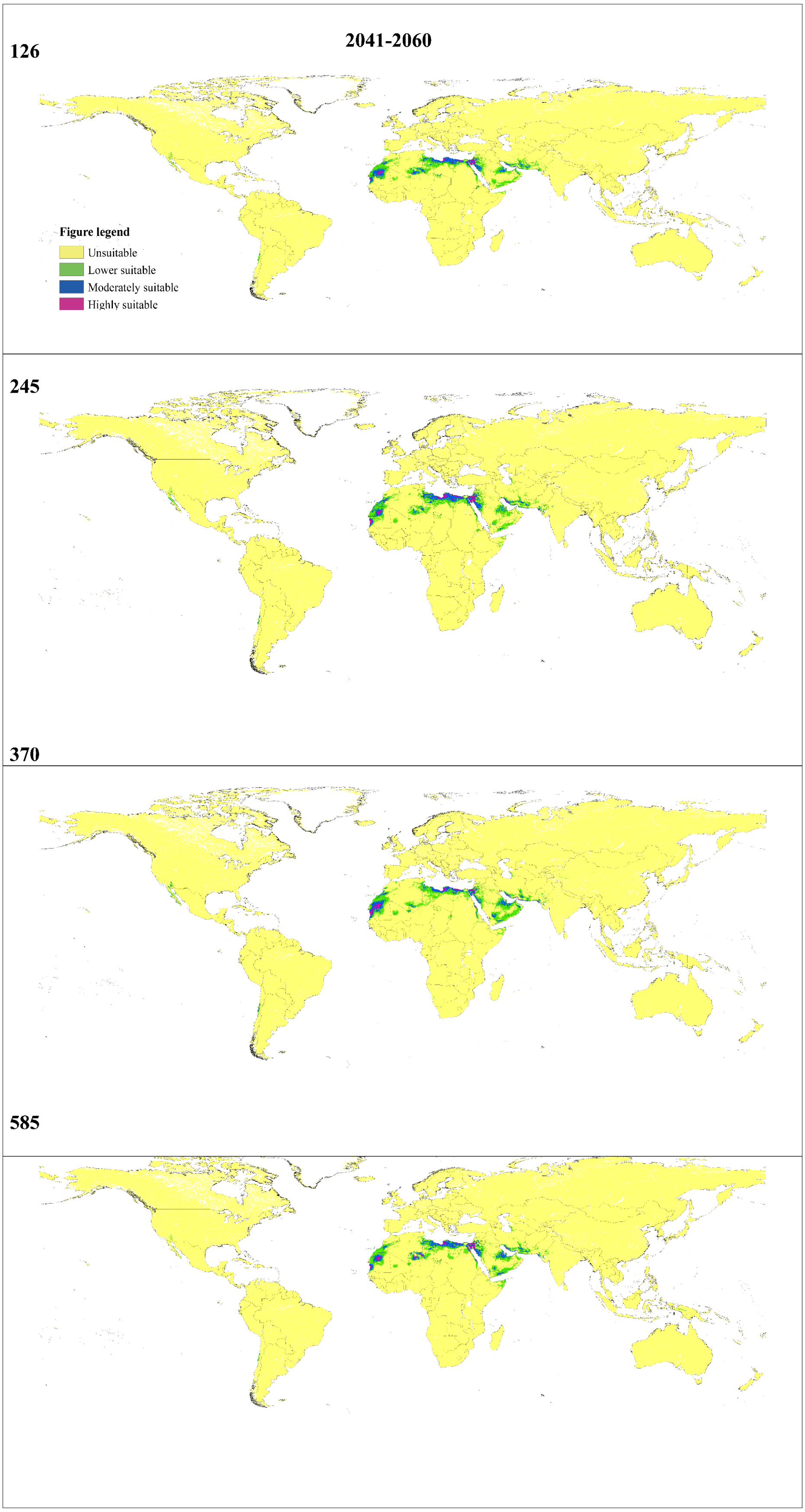

Figure 16s. Potential distribution of *Anastatica hierochuntica* in 2041-2060.

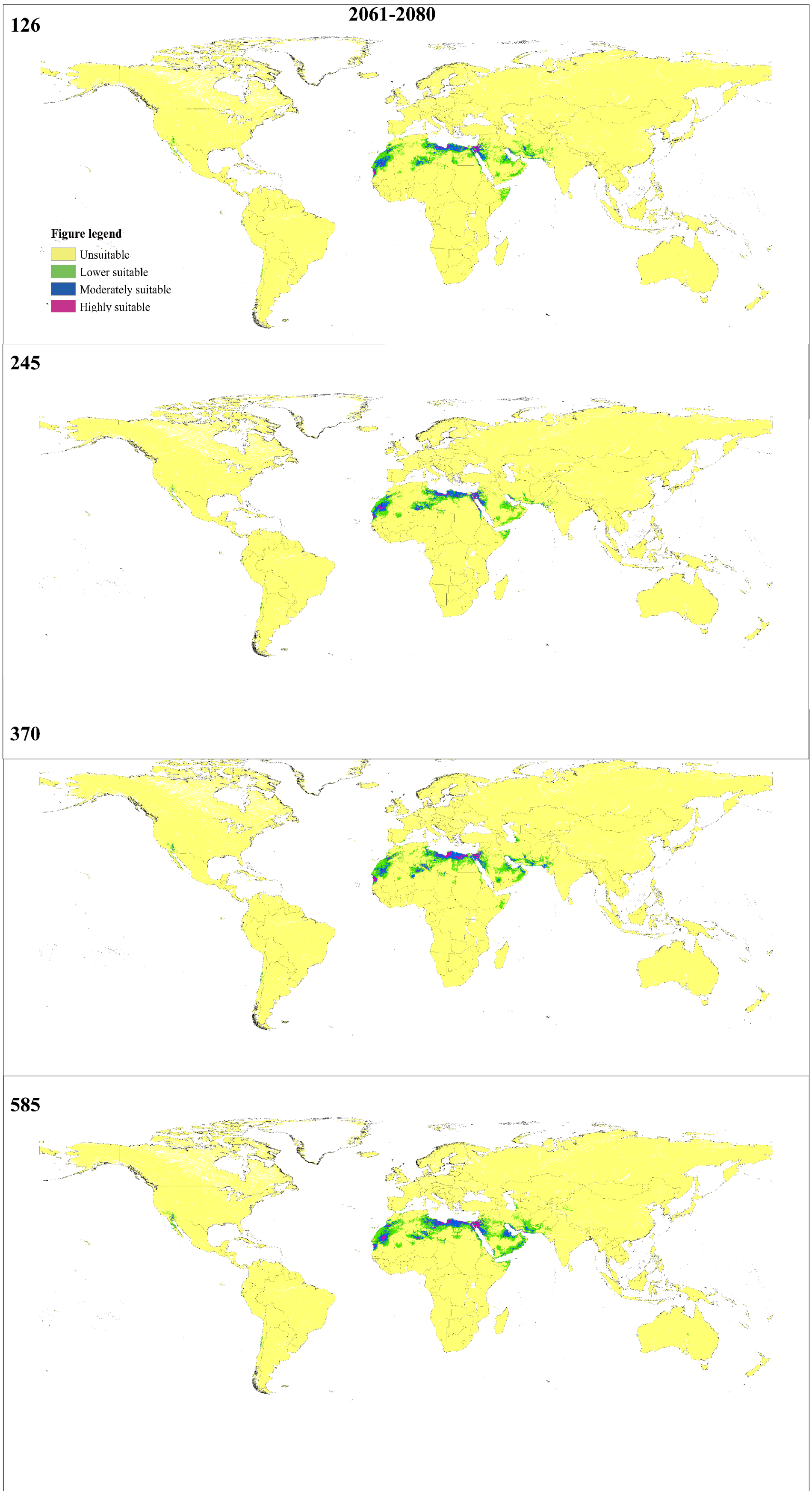

Figure 17s. Potential distribution of *Anastatica hierochuntica* in 2061-2080.

2081-2100

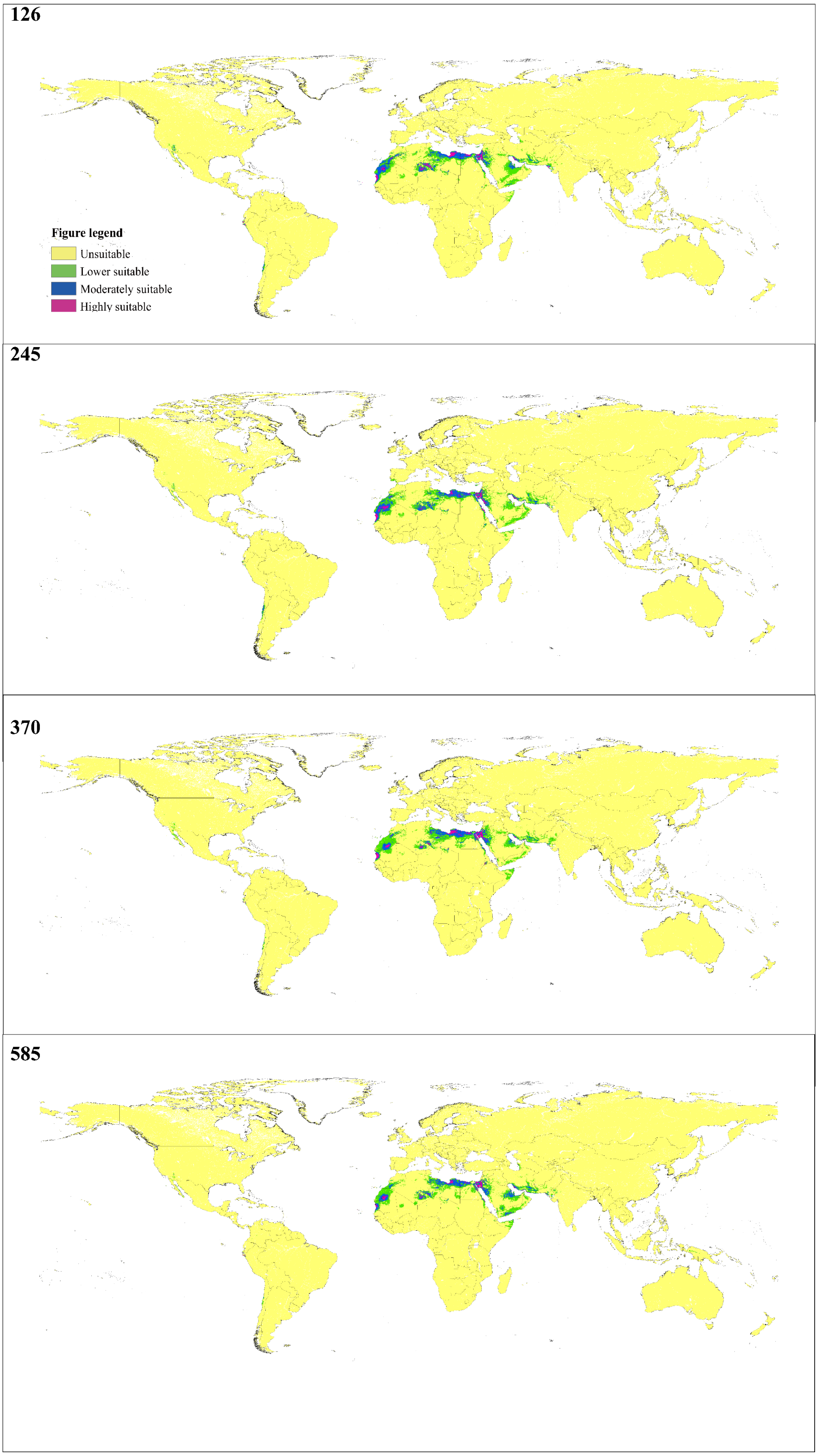

Figure 18s. Potential distribution of *Anastatica hierochuntica* in 2081-2100.

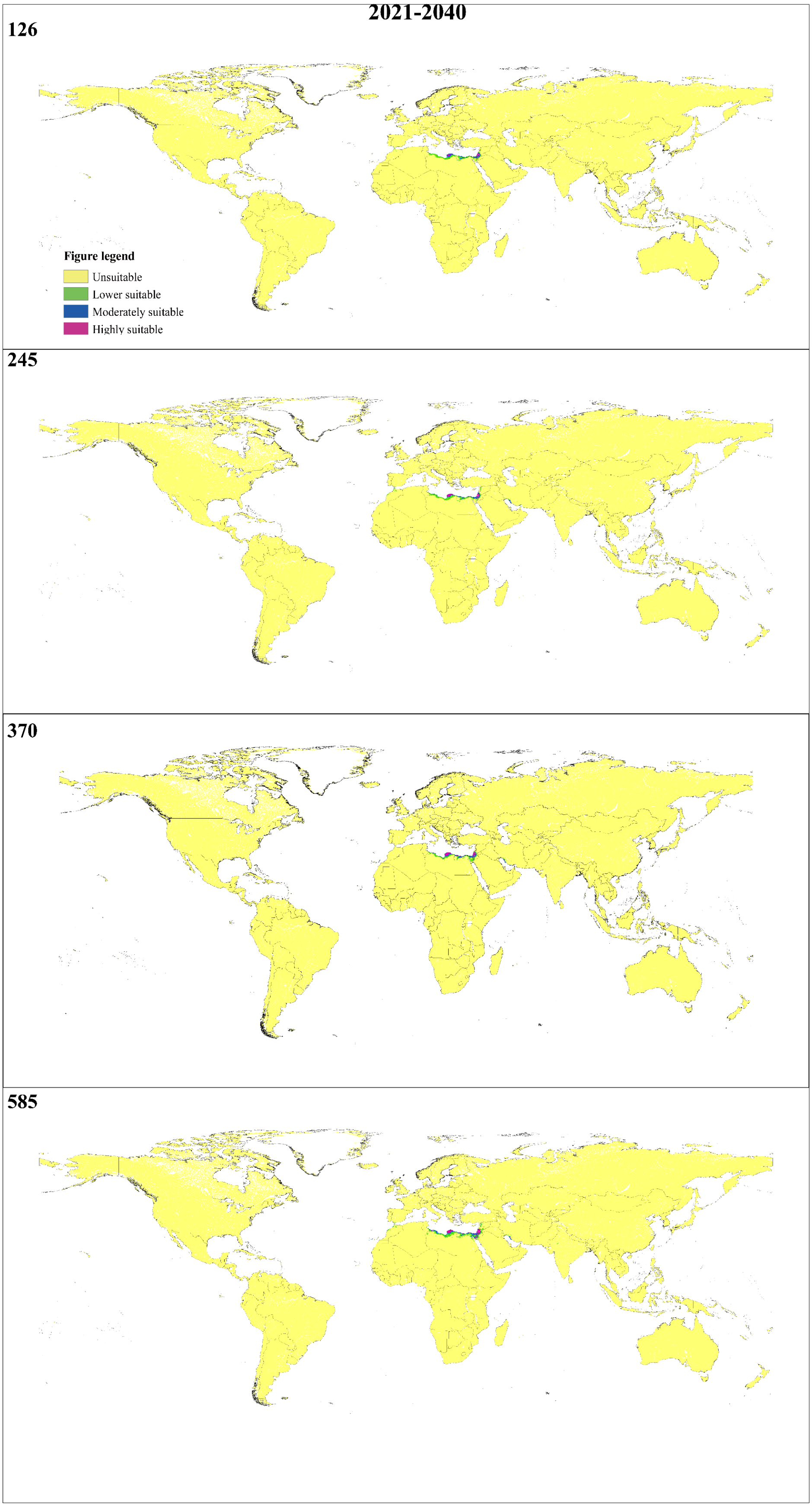

Figure 19s. Potential distribution of *Trigonella arabica* in 2021-2040.

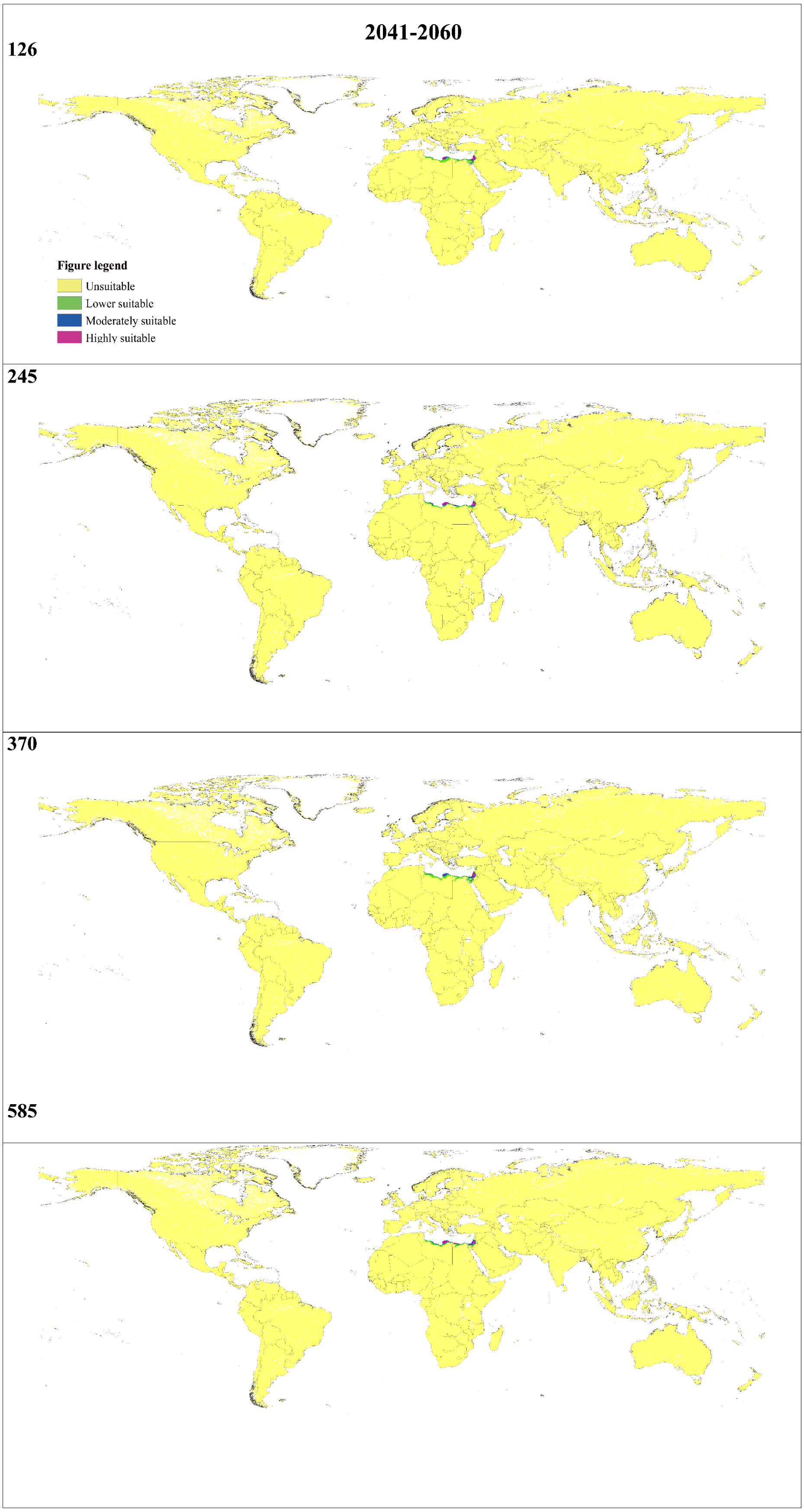

Figure 20s. Potential distribution of *Trigonella arabica* in 2041-2060.

2061-2080

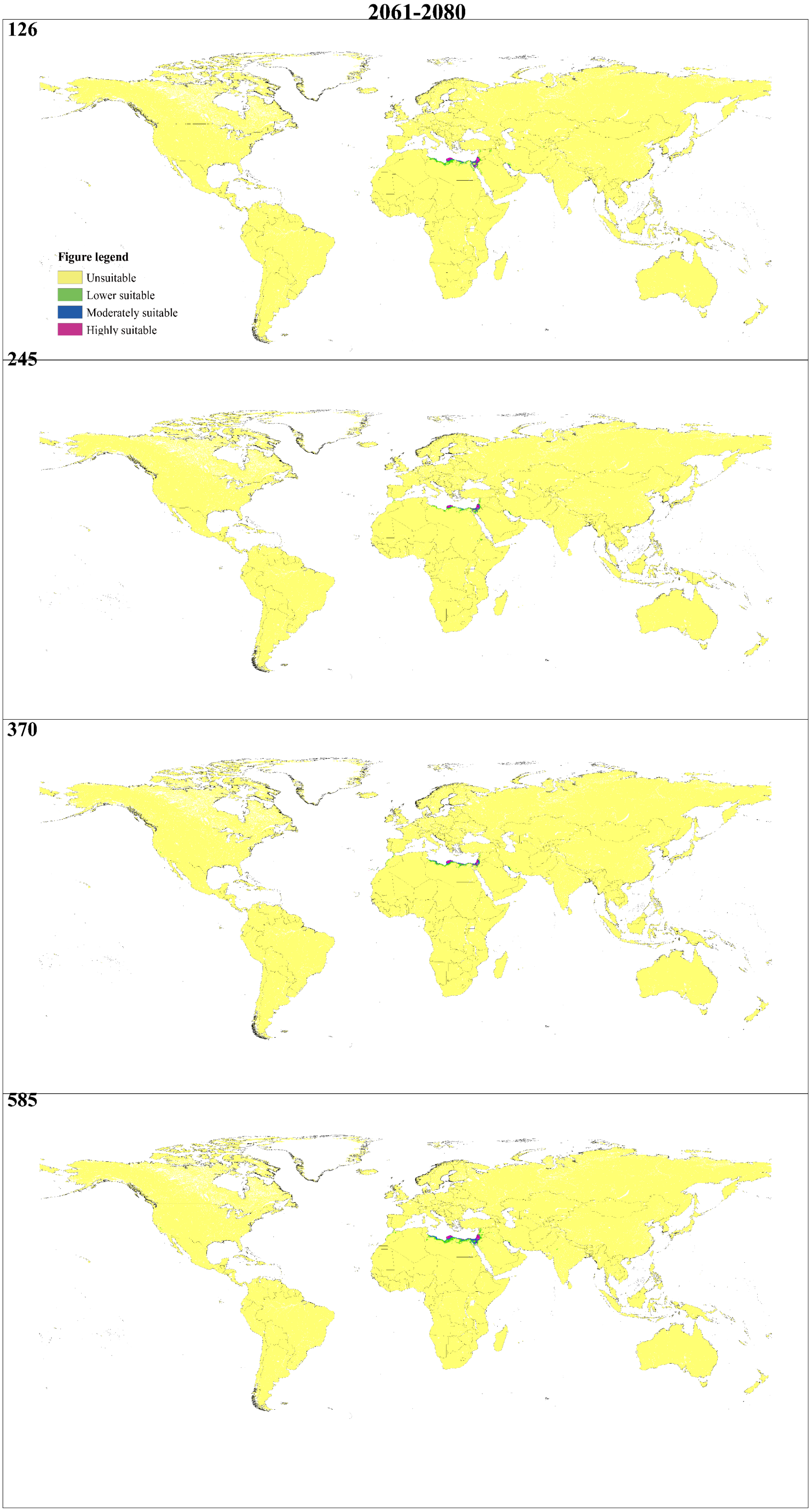

Figure 21s. Potential distribution of *Trigonella arabica* in 2061-2080.

2081-2100

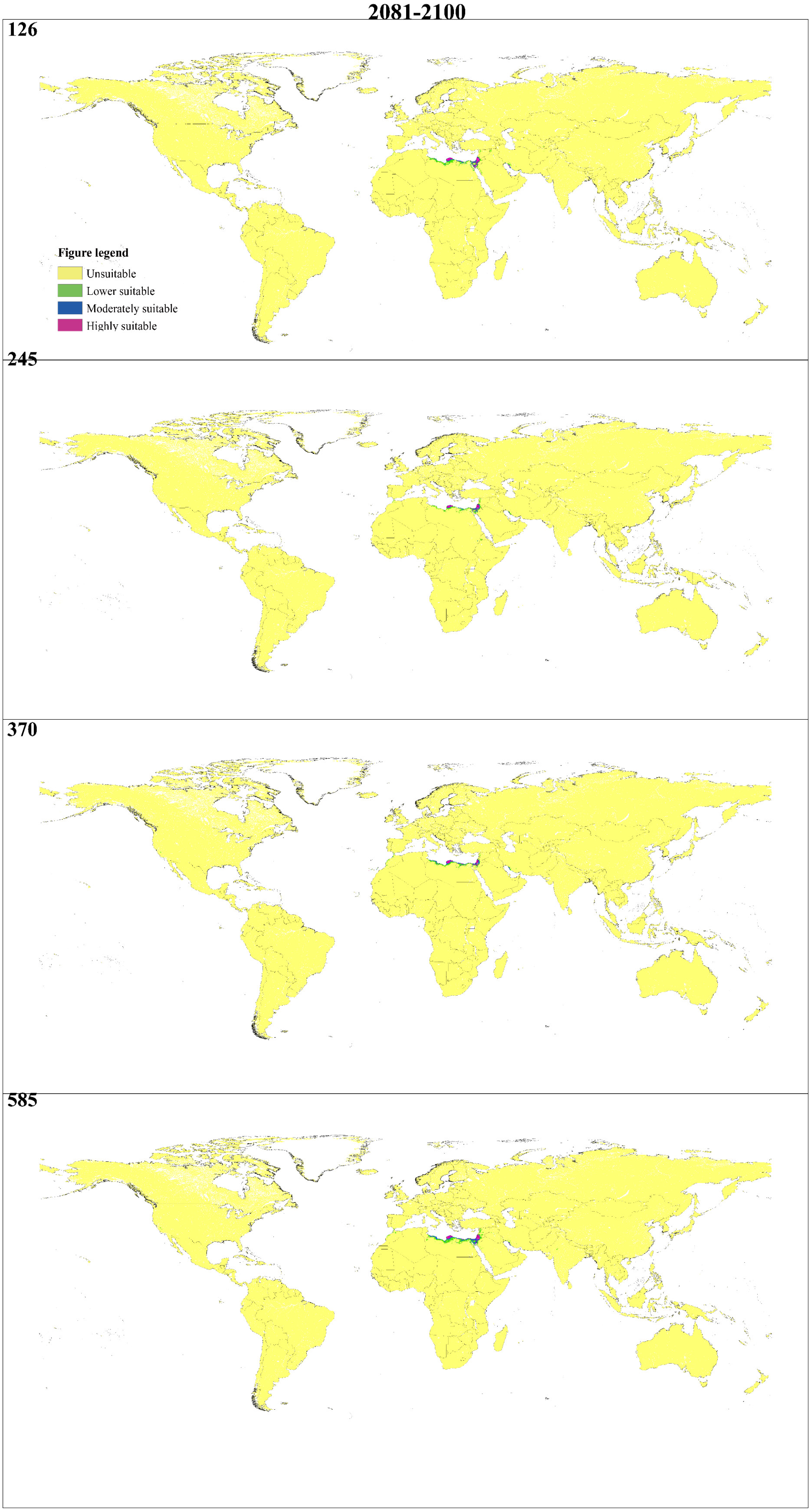

Figure 22s. Potential distribution of *Trigonella arabica* in 2081-2100.

Deciduous forest

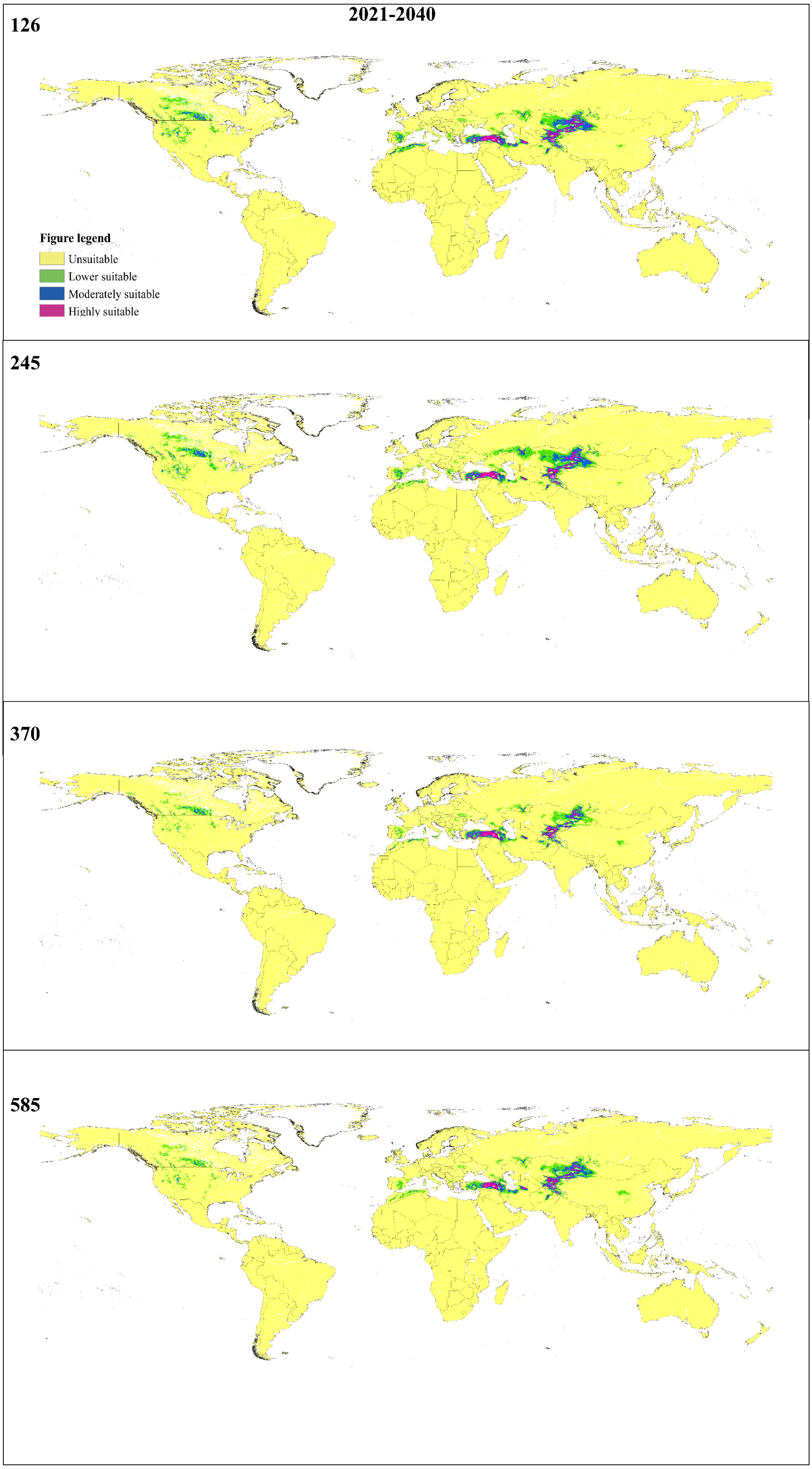

Figure 23s. Potential distribution of *Gagea filiformis* in 2021-2040.

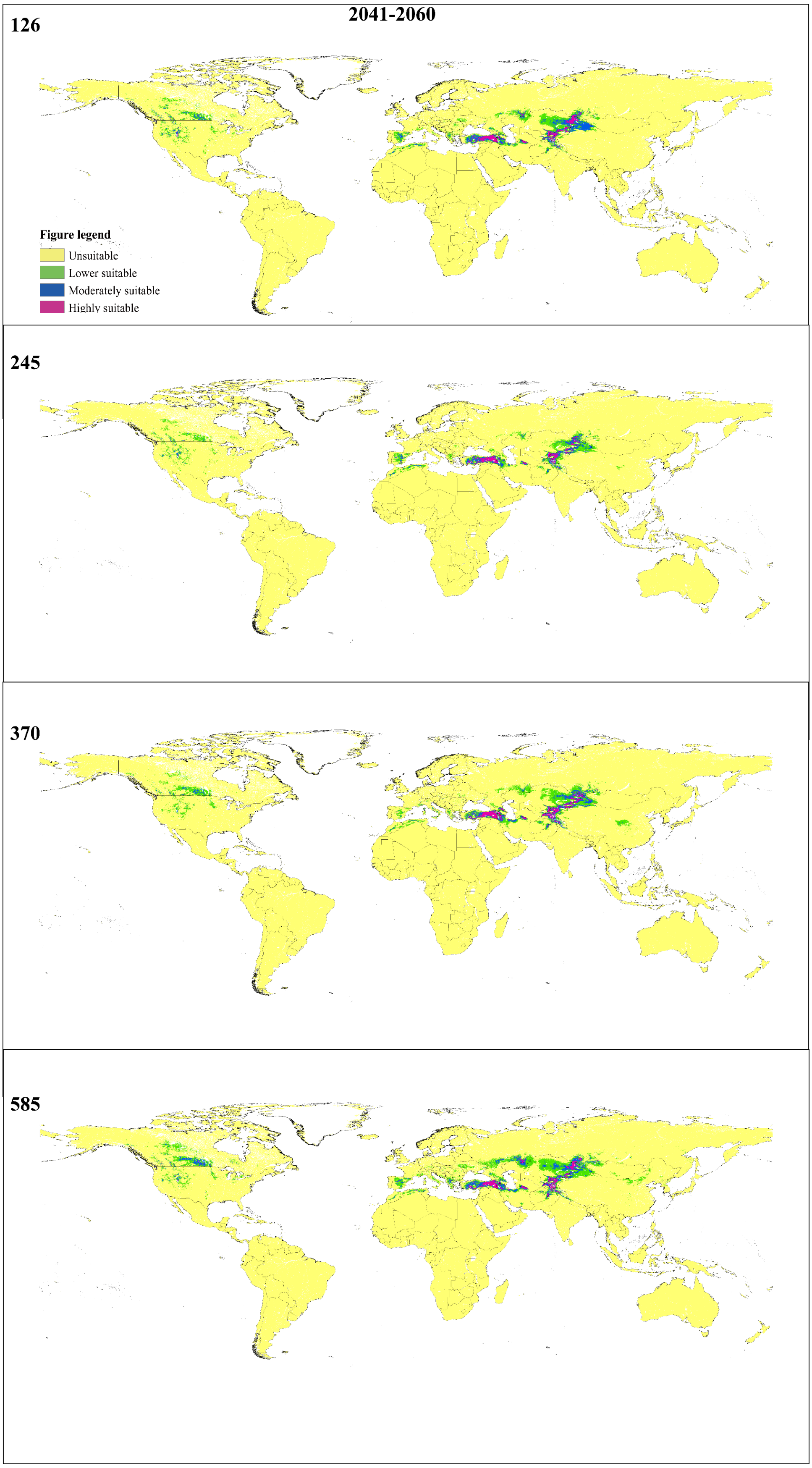

Figure 24s. Potential distribution of *Gagea filiformis* in 2041-2060.

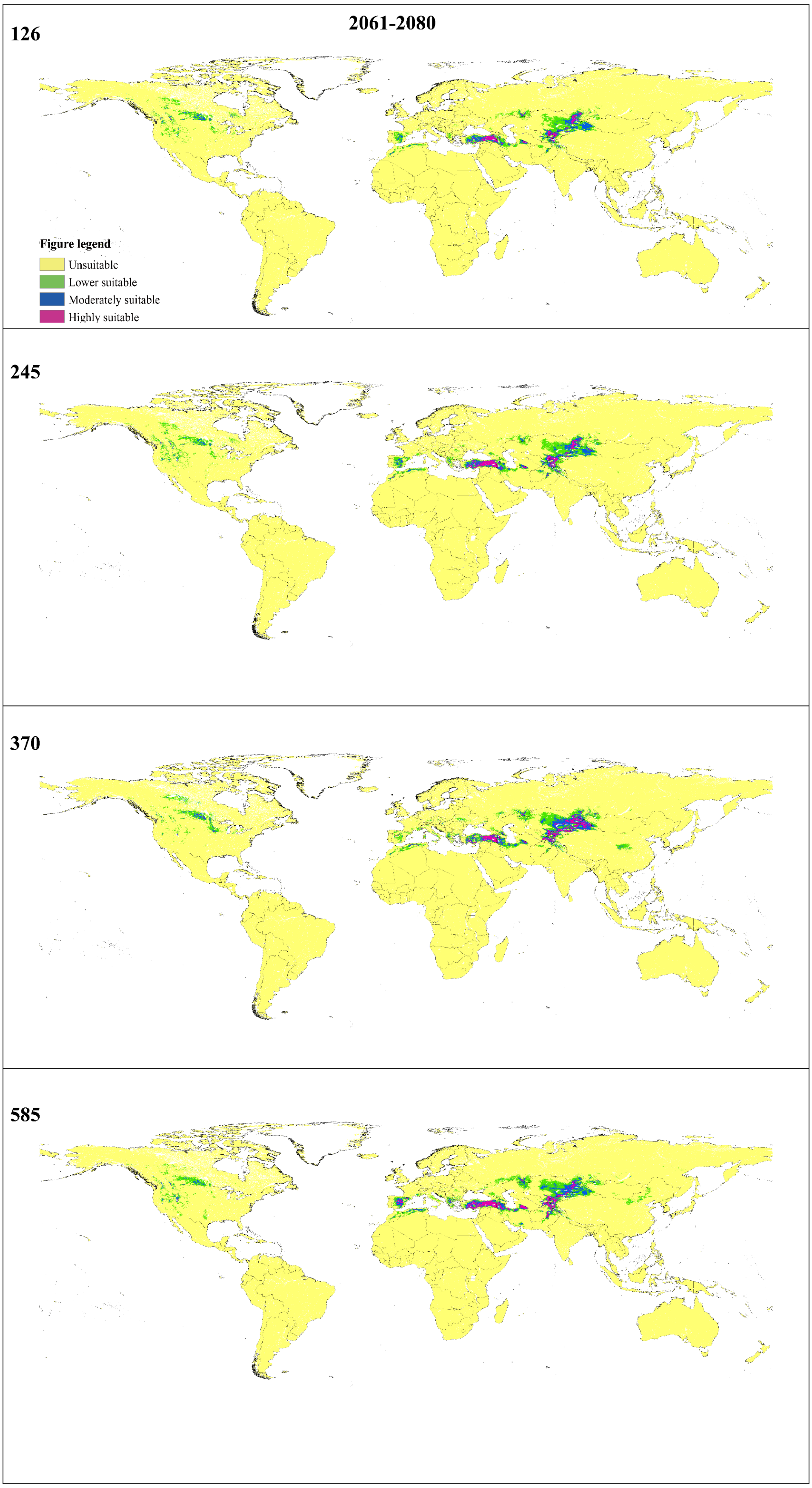

Figure 25s. Potential distribution of *Gagea filiformis* in 2061-2080.

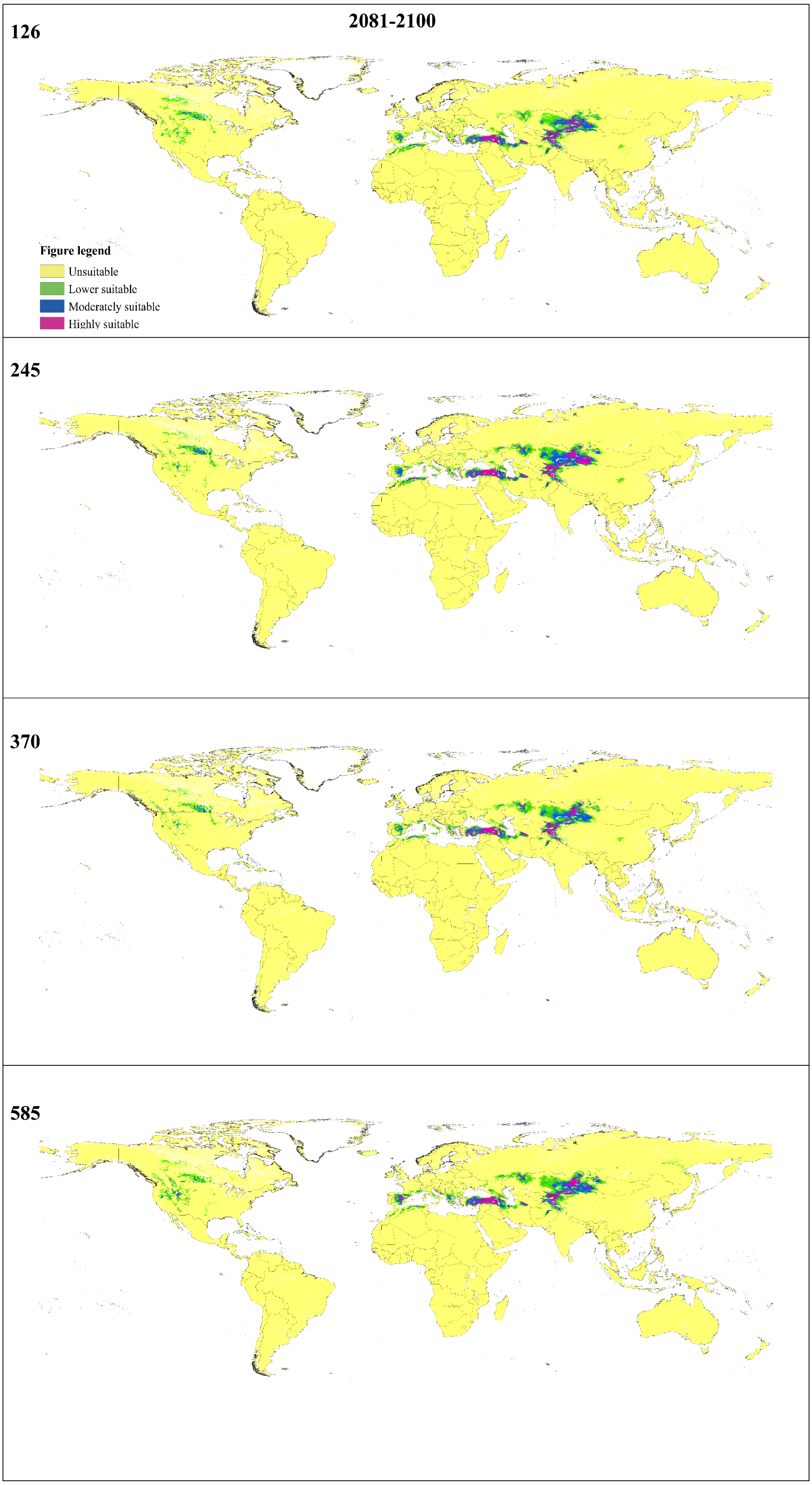

Figure 26s. Potential distribution of *Gagea filiformis* in 2081-2100.

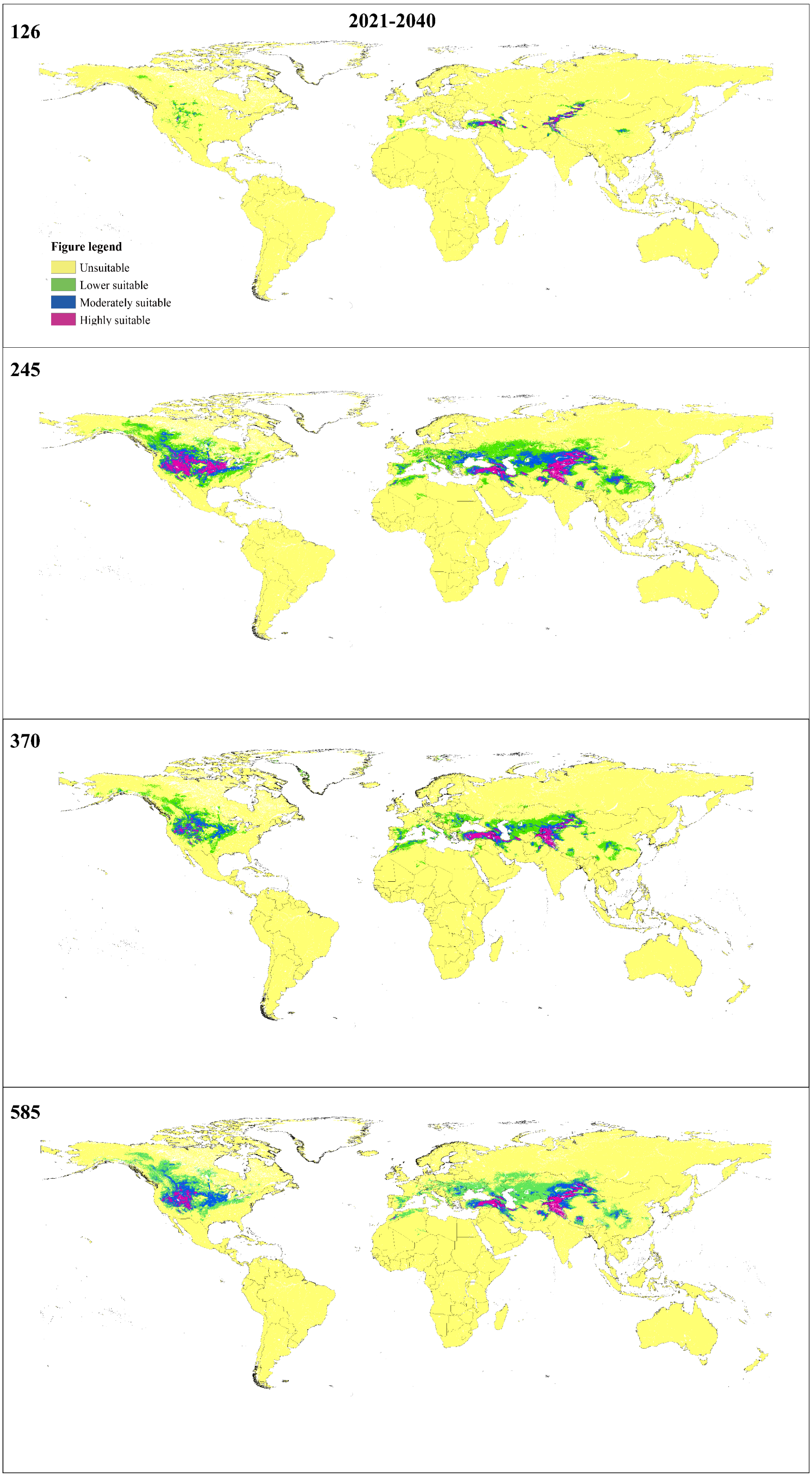

Figure 27s. Potential distribution of *Crocus alatavicus* in 2021-2040.

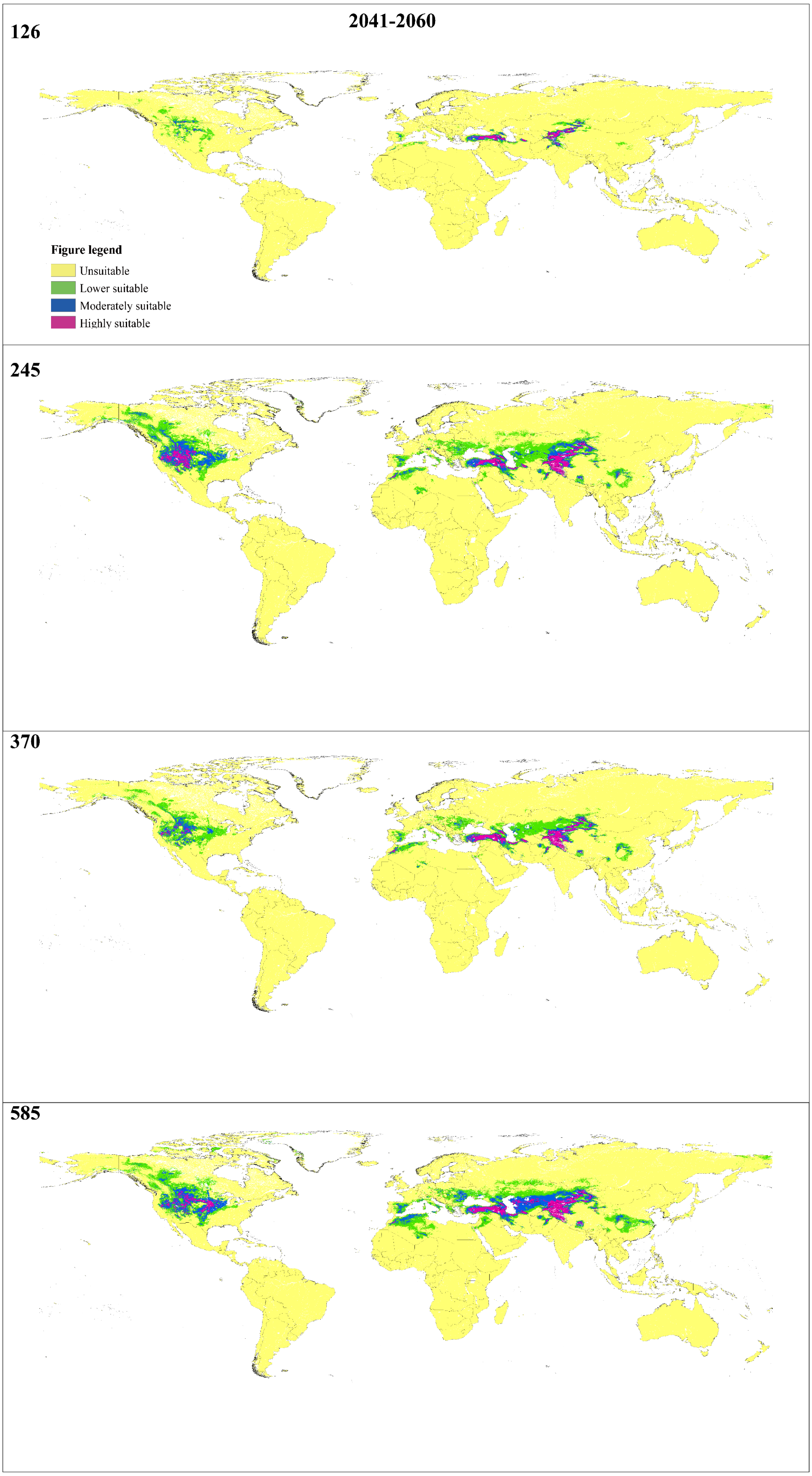

Figure 28s. Potential distribution of *Crocus alatavicus* in 2041-2060.

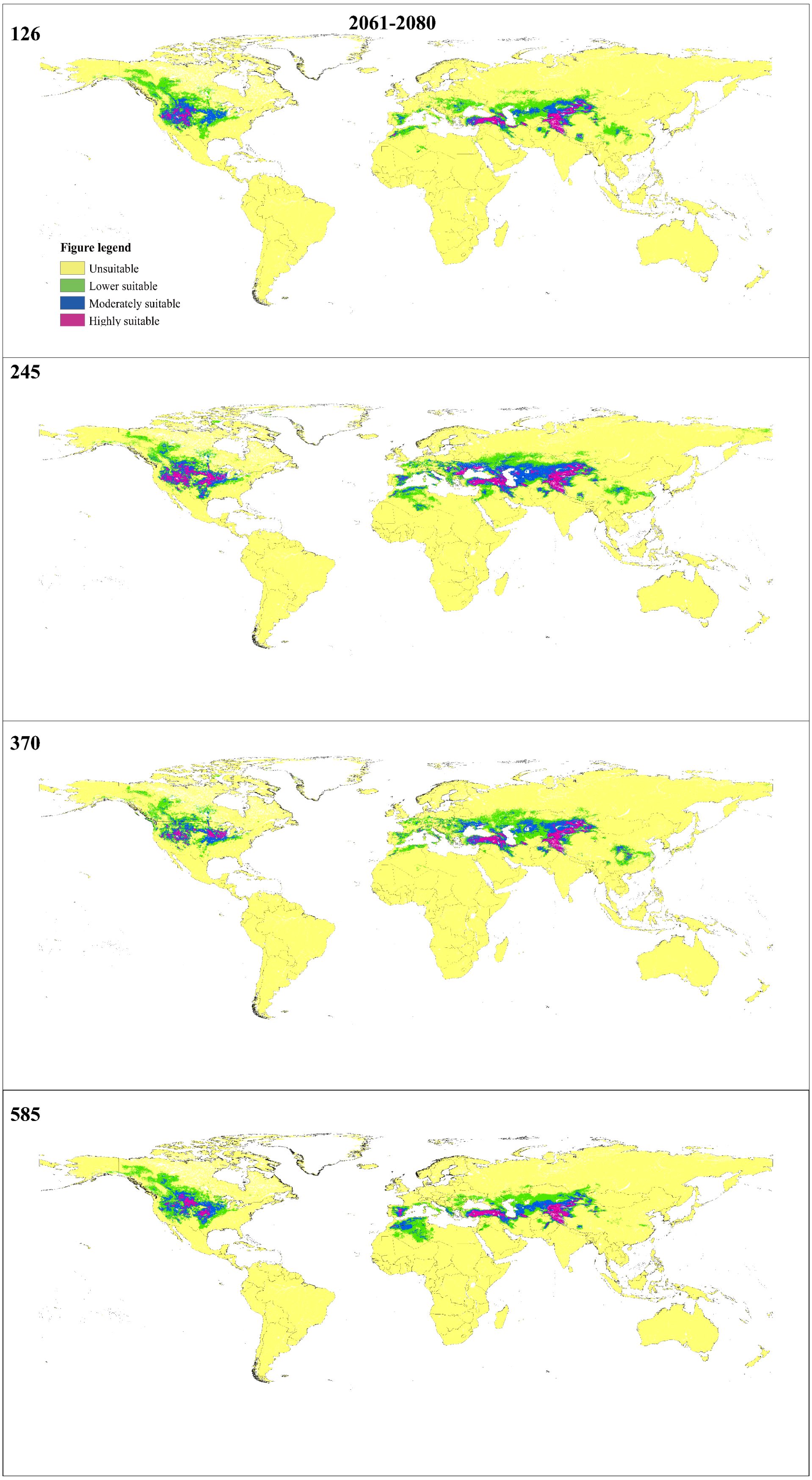

Figure 29s. Potential distribution of *Crocus alatavicus* in 2061-2080.

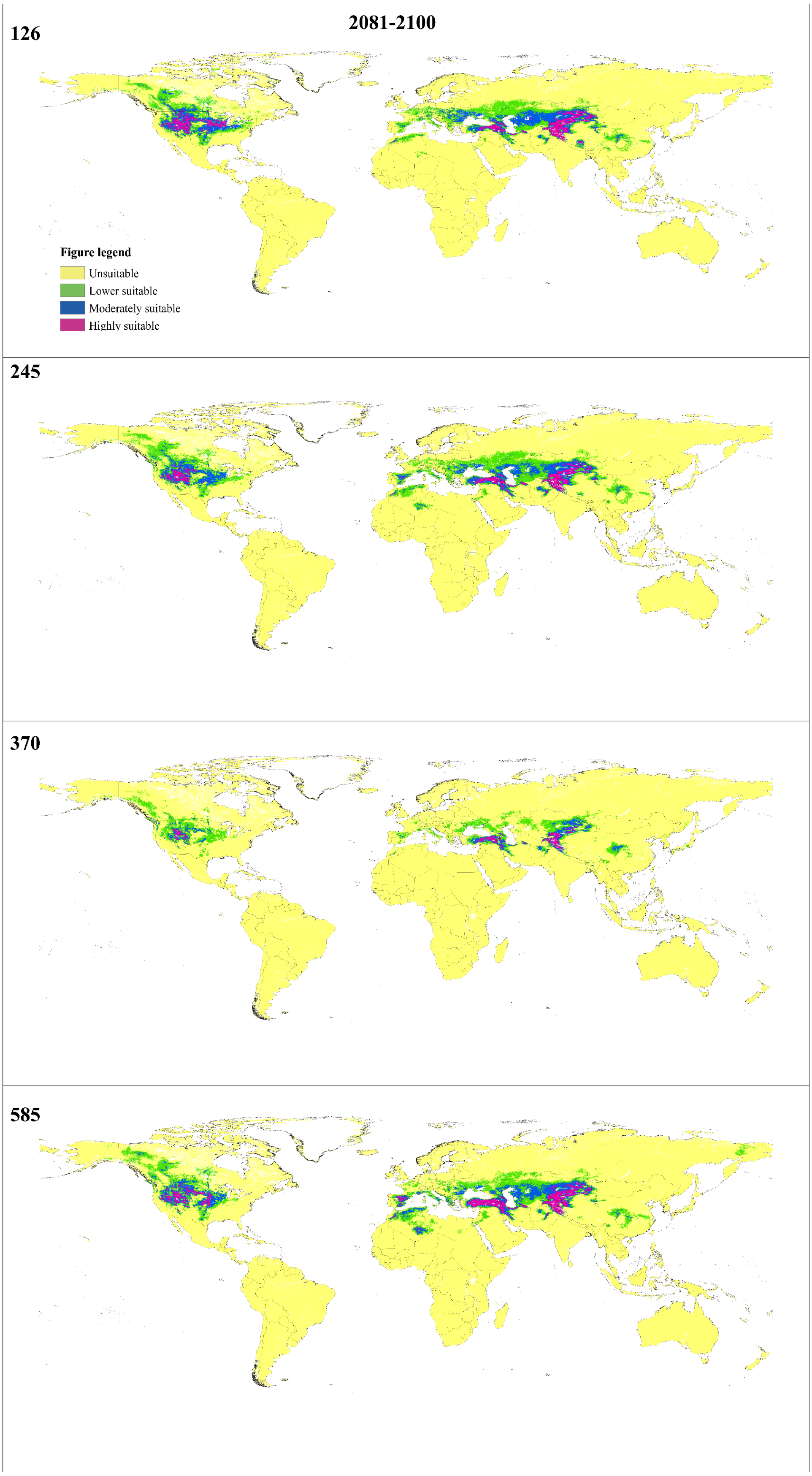

Figure 30s. Potential distribution of *Crocus alatavicus* in 2081-2100.
